# A fingerprint-based polymeric sensing platform for comprehensive quality assessment of complex culture media in cell manufacturing

**DOI:** 10.1039/d6sc00383d

**Published:** 2026-04-17

**Authors:** Shunsuke Tomita, Kumi Morikawa, Naoshi Kojima, Sayaka Ishihara, Hiroyuki Kusada, Hideyuki Tamaki, Ryoji Kurita

**Affiliations:** a Health and Medical Research Institute, National Institute of Advanced Industrial Science and Technology (AIST) 1-1-1 Higashi Tsukuba Ibaraki 305-8566 Japan s.tomita@aist.go.jp; b School of Integrative and Global Majors, University of Tsukuba 1-1-1 Tennodai Tsukuba Ibaraki 305-8577 Japan; c Health and Medical Research Institute, AIST 2217-14 Hayashi-cho Takamatsu Kagawa 761-0301 Japan; d Biomanufacturing Process Research Center, AIST 1-1-1 Higashi Tsukuba Ibaraki 305-8566 Japan; e Faculty of Pure and Applied Sciences, University of Tsukuba 1-1-1 Tennodai Tsukuba Ibaraki 305-8573 Japan

## Abstract

The rapid advancement of cell manufacturing across biotechnology, regenerative medicine, and cellular agriculture is driving a growing demand for simple and reliable analytical tools to ensure the consistent quality of complex culture media, whether derived from natural or synthetic sources. Here, we present a hypothesis-free, data-driven polymeric sensing platform that employs an array of synthetic polymer probes to generate fluorescence-response fingerprints, enabling the statistical detection of subtle compositional differences in complex biological mixtures. This approach, based on charged block-copolymers conjugated with aggregation-induced-emission (AIE) fluorophores, successfully distinguishes 16 animal sera and identifies differences in serum origin, lot, and storage conditions through rapid and simple fluorescence measurements. Unexpectedly, the resulting response fingerprints also encode phylogenetically informative signals among animal species. Furthermore, the platform detects quality variations in serum-free supplements for stem-cell culture and naturally derived supplements used for microbial culture, including subtle compositional changes undetectable by standard cell-culture assays. As this fingerprint-based strategy does not require prior assumptions about which specific components are important, it can be flexibly adapted to a diverse array of supplement types and quality control needs. Overall, this versatile sensing platform provides a robust and reproducible framework for proactive quality assessment in cell manufacturing, supporting the reliable production of cell-based products.

## Abstract 2

A hypothesis-free polymeric sensor array generates fluorescence fingerprints to rapidly identify the type, origin, and quality of complex culture supplements, enabling robust and data-driven quality assessment across diverse biological media.

## Graphical abstract

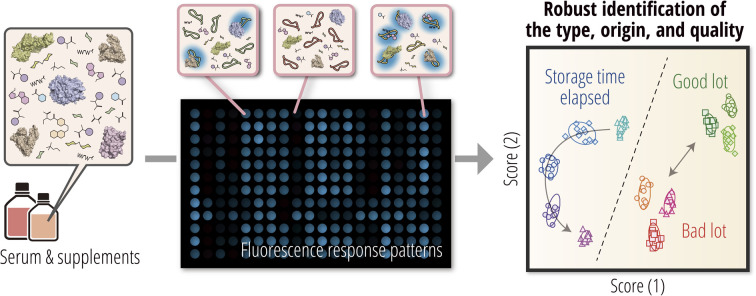

## Introduction

With the rapid advancement of biotechnology, cell-culture technology has extended its applications beyond diagnostics, drug discovery, and regenerative medicine^1–3^ to include cellular agriculture, such as cultured-meat production.^4,5^ This expansion positions cell culture as an essential technology for the realization of a sustainable circular bioeconomy without excessive dependence on fossil-fuel resources.^6,7^ To realize the diverse applications of cell culture, the appropriate selection of the culture media is critical, as it directly affects cell proliferation, viability, and phenotype. Traditionally, serum, particularly fetal bovine serum (FBS), has been widely used as a supplement due to its high content of essential components such as growth factors, cytokines, and vitamins. However, concerns over lot heterogeneity, animal welfare, and supply sustainability have led to significant efforts to develop serum alternatives. While these alternatives aim to achieve chemically defined formulations, they often fall short in replicating the full biological functionality of conventional serum. As a result, biologically derived supplements such as human platelet lysate and silk-derived sericin proteins are receiving increasing attention.^8–11^ In cellular agriculture, plant- and microbe-derived extracts or hydrolysates are particularly valued for their low cost and compatibility with food-grade standards,^12^ while their potential applications in biopharmaceutical manufacturing are also under investigation.^13,14^

Cell-culture supplements play a crucial role in determining culture success and thus require strict quality control. The quality of a cell-culture supplement is influenced not only by the harvesting and processing used in its manufacture, but also by factors such as the origin of the raw materials, the infrastructure of the manufacturing company, and variations in handling and regulation.^9,10^ These factors contribute to significant batch-to-batch variability, posing a major challenge to achieving consistent performance when using a supplement to support cell culture.^15,16^ In practice, the information provided by suppliers is often limited to partial protein content, results from endotoxin and viral testing, or growth assays using specific cell lines. Therefore, end users are typically required to perform their own lot-to-lot verification by culturing cells, which is both time- and labor-intensive.^15,16^ Furthermore, such verification is frequently affected by the initial condition of the cells and the skill level of the operator, making it difficult to ensure consistent and reliable quality assessment across batches. Conventional analytical techniques such as immunoassays or chromatographic analyses can quantify specific components but provide limited insight into the overall biochemical composition of complex supplements. Similarly, fluorescence-based sensing strategies, although offering high sensitivity, rely on highly specific molecular recognition and therefore provide only partial information about the overall composition of such complex mixtures.

To address these quality-assessment challenges, chemical-sensing approaches that couple multivariate data acquisition with statistical analysis offer a promising solution. These sensing approaches, commonly referred to as chemical nose/tongue systems or pattern-based sensor arrays,^17–20^ differ fundamentally from conventional sensing methods that rely on specific molecular recognition by antibodies or enzymes. Instead, the arrays emulate the mammalian olfactory and gustatory systems by leveraging a diverse set of cross-responsive, non-specific interactions. Rather than targeting a single molecule, the arrays are designed to extract comprehensive compositional information from complex samples. Therefore, this approach is also described as a “hypothesis-free sensor array”, a term that reflects the ability of the array to detect differences without prior assumptions about which specific components are important.^21,22^ These characteristics make hypothesis-free sensor arrays particularly well suited for the quality assessment of complex culture media, in which performance-critical components are often unknown, ill-defined, or highly variable.

In a typical case, these systems employ a set of environmentally responsive optical probes with structurally diverse properties. Upon interaction with the target sample, the probe array generates a distinct optical response fingerprint that reflects the sample's overall composition. Processing these fingerprints through multivariate analysis or machine learning enables accurate classification and identification of the composition of complex biological samples, such as cells,^23–28^ microorganisms,^29–35^ and fermented beverages.^21,36,37^ These methods have also been applied to human serum, where they have been shown capable of distinguishing between healthy individuals and unwell patients.^38–41^ We have further demonstrated that this approach can, through the analysis of culture supernatants, non-invasively detect cellular phenotypes, including stem-cell differentiation,^42,43^ fibroblast senescence,^44^ and drug responses in lung-cancer cells.^45,46^

**Fig. 1:**
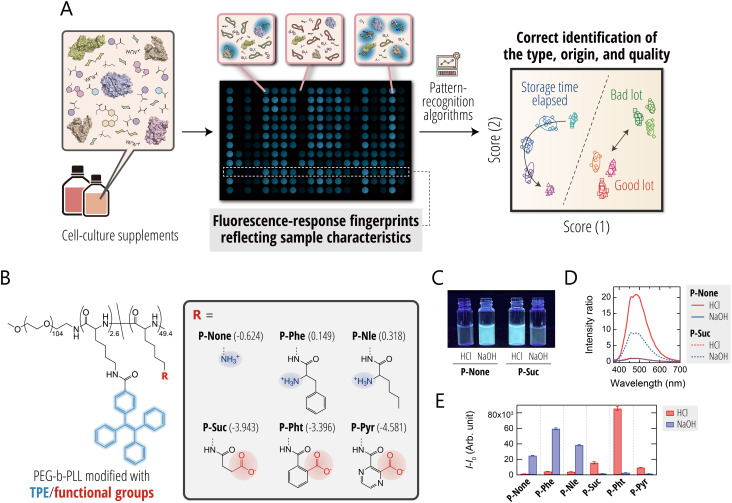
(A) Schematic representation of how the hypothesis-free polymeric sensor platform is used for the optical quality assessment of cell-culture supplements. Cell-culture supplements are mixed with AIE-polymers in an array format, and the resulting fluorescence-response fingerprints are analyzed using statistical techniques to determine the type, origin, and quality of the supplement. (B) Molecular structures of environmentally responsive AIE-polymers. The log *P* values of the head groups are shown in parentheses. (C) Photographs of P-None and P-Suc solutions (10.0 µM) in 10 mM HCl or 10 mM NaOH under UV illumination. (D) Fluorescence spectra and (E) fluorescence-intensity changes of AIE-polymers (2.0 µM) in 10 mM HCl or 10 mM NaOH; *λ*_ex_ = 330 nm for (D) and *λ*_ex_/*λ*_em_ = 330 nm/480 nm for (E). The values represent mean ± 1 SE values from three independent experiments.

In this study, we applied a hypothesis-free sensing strategy to assess the quality of complex culture media and supplements used in cell manufacturing (Fig. 1A). Focusing on serum and its alternatives, we constructed sensor arrays composed of aqueous solutions of diverse charged synthetic polymer probes that exhibit aggregation-induced emission (AIE). Statistical analysis of the resulting fluorescence-response fingerprints enabled an accurate classification of supplements according to type, origin, and quality. Beyond conventional quality metrics, the sensing platform captured subtle compositional variations associated with lot differences, storage conditions, and degradation processes, including changes that were not detected by standard cell-based assays. Unexpectedly, the response fingerprints also encoded phylogenetic relationships among animal species, indicating that the hypothesis-free sensing approach can extract biologically meaningful information beyond predefined quality parameters. Together, this hypothesis-free, data-driven polymeric sensing platform offers a robust and reproducible framework for early and comprehensive quality assessment of complex culture media. By enabling proactive detection of quality variation without prior assumptions about critical components, this approach has the potential to support standardization, reduce manufacturing variability, and improve the reliability of cell-based product manufacturing across diverse applications.

## Results and discussion

### Design of the sensing platform

To comprehensively capture optical information reflecting the complex composition of cell-culture supplements, such as serum and its alternatives, which contain a broad spectrum of components including proteins (*e.g.*, albumin, growth factors, and cytokines), amino acids, sugars, and vitamins – a sensor framework capable of interacting with a variety of molecular species is required. Charged synthetic polymers are well-suited for this purpose, as they engage with a wide range of biomolecules across different structural hierarchies.^22,43,46–48^ In particular, poly-l-lysine (PLL), which is rich in primary amino groups, can be easily functionalized *via* nucleophilic chemistry.^20,49^

In this study, we selected a block copolymer of poly(ethylene glycol)-*block*-PLL (PEG-*b*-PLL) as a scaffold capable of enhancing aqueous dispersibility and suppressing precipitation. Using this scaffold, we introduced: (i) a tetraphenylethylene (TPE) moiety with AIE properties, low background fluorescence, and high sensitivity and (ii) a chemically diverse set of functional groups varying in charge, hydrophobicity, and π–π stacking capacity (Fig. 1B).^34^

TPE is a representative AIE fluorophore that is essentially non-emissive in the dispersed state because intramolecular rotations promote nonradiative decay, but becomes highly emissive when molecular motion is restricted, *e.g.*, through aggregation or binding-induced restriction of intramolecular motion (RIM).^50^ This sharp turn-on-fluorescence response is widely exploited for sensitive sensing applications.^51^

Additional functional groups were introduced onto the residual amino groups of P-None, a polymer partially modified with TPE, to introduce diverse interaction capabilities toward the biomolecular components present in cell-culture supplements. Specifically, the remaining amino groups were modified with (i) amino-acid-derived substituents possessing different aromatic and hydrophobic properties (P-Phe and P-Nle) or (ii) acid anhydrides with varying hydrophobicity in order to induce charge inversion from cationic to anionic polymers (P-Suc, P-Pht, and P-Pyr). Through this design, the resulting polymers exhibit diverse physicochemical properties, including variations in charge, hydrophobicity, and aromatic interaction potential.

To confirm that the TPE moieties in these polymers exhibit AIE characteristics, we examined fluorescence responses consistent with the RIM mechanism (Fig. 1C–E). When the charge state of the polymers was varied *via* the pH value, the cationic polymers (P-None, P-Phe, and P-Nle) showed very weak fluorescence under acidic conditions but pronounced fluorescence enhancement under basic conditions. In contrast, the anionic polymers (P-Suc, P-Pht, and P-Pyr) displayed the opposite trend. This behavior likely arises from increased intermolecular association upon charge neutralization. Such charge-neutralization-induced association is widely reported for polyelectrolytes^52^ and may reduce electrostatic repulsion and restrict molecular motion, leading to fluorescence enhancement.

To further examine whether this fluorescence enhancement is related to RIM rather than direct charge effects, we investigated the influence of viscosity (Fig. S1). Increasing glycerol concentration under conditions where the polymers were initially non-emissive led to viscosity-dependent fluorescence enhancement for all polymers. These results are consistent with the RIM mechanism widely reported for TPE-based AIE luminogens.^50,53^

Based on this design and the above observations, we expected that this array of structurally diverse AIE-polymers would differentially interact with the constituent biomolecules in the supplements, converting subtle compositional differences into distinct AIE fluorescence-response fingerprints that enable accurate assessment of the supplement composition.

### Identification of animal species

**Fig. 2:**
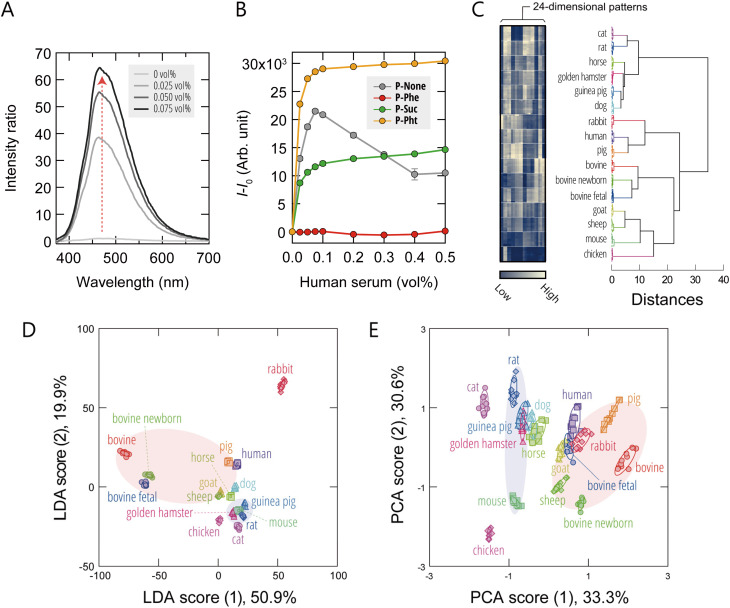
Characterization of the representative AIE-polymers and the optical classification of sera from various animal species. (A) Fluorescence spectra of P-None (300 nM) upon addition of human serum (0–0.075 vol%) in 20 mM MOPS buffer (pH = 7.0); *λ*_ex_ = 330 nm. (B) Binding isotherms for different AIE-polymers (300 nM) upon addition of human serum in 20 mM MOPS buffer (pH = 7.0); *λ*_ex_/*λ*_em_ = 330 nm/480 nm. The values shown represent mean values ± 1 SE from three independent experiments. (C) Heatmap and corresponding HCA dendrogram of the fluorescence-response fingerprints of the 16 different sera (0.10 vol%). For each analyte, ten independent experimental values are shown. Each column in the heatmap corresponds to the terminal branch of the dendrogram. (D) LDA score plot and (E) PCA score plot for the sera. Ellipsoids represent the confidence intervals (±1 SD) for each group. In (D) and (E), the red and blue translucent ellipsoids indicate meta-clusters corresponding to the taxonomic orders Artiodactyla and Rodentia, respectively.

We first evaluated the responsiveness of the AIE-polymers to human serum. When human serum was added to P-None at a polymer concentration of 300 nM, the fluorescence intensity at the emission peak (*λ*_ex_/*λ*_em_ = 330 nm/460 nm) exhibited a non-monotonic response, reaching a 65-fold enhancement at 0.075 vol% (Fig. 2A), before decreasing at higher concentrations (Fig. 2B). This nonlinear behavior suggests that multiple serum components with different affinities and fluorescence-enhancing capabilities compete to bind with the polymer, potentially leading to changes in aggregation states or binding configurations at higher concentrations. Notably, P-Phe (a polymer with hydrophobic phenyl groups) exhibited little fluorescence change even at 0.500 vol%, whereas the anionic polymers P-Suc (without aromatic groups) and P-Pht (with aromatic groups) showed pronounced signal enhancement, with their responses saturating at ∼0.100 vol% (Fig. 2B). These results collectively demonstrate that the AIE-polymers bind with components in human serum through various modes, thereby capturing the multifaceted nature of the serum composition.

For completeness, absorption and fluorescence spectra of the AIE-polymers (2.0 µM) before and after addition of human serum are provided in Fig. S2 and S3. A pronounced fluorescence enhancement was observed upon serum addition for all polymers.

Having confirmed the responsiveness of the AIE-polymers to serum, we next used sera derived from various animal species as a model system for cell-culture supplements to test whether or not the sensor array could generate unique fluorescence-response fingerprints for these samples. The sensing procedure was as follows: each serum sample was added to an array composed of six AIE-polymers (300 nM) dissolved in either 20 mM MOPS buffer (pH = 7.0) or 20 mM acetate buffer (pH = 5.0). We employed two different pH conditions to modulate the charge states of both serum components and polymers, thereby enabling the extraction of compositional information that might not be accessible under a single pH condition. For each serum/probe combination, fluorescence responses were recorded using two independent detection channels (Ch1: *λ*_ex_/*λ*_em_ = 330 nm/480 nm; Ch2: *λ*_ex_/*λ*_em_ = 360 nm/530 nm), yielding 24-dimensional fluorescence-response fingerprints (6 polymers × 2 pH values × 2 channels).

A visual summary of the response fingerprints in the form of heatmaps (Fig. 2C and S4A; raw data available in Dataset S1) revealed that a series of diverse and distinctive fluorescence profiles were generated upon addition of serum. Unsupervised hierarchical cluster analysis (HCA), which groups samples based on fingerprint similarity without prior knowledge of sample identity, segregated the fluorescence responses into distinct clusters, with each cluster corresponding to a specific animal species (Fig. 2C). These results demonstrate that the differences in response fingerprints between species were both reproducible and statistically significant.

To assess the discriminative power of our hypothesis-free polymeric sensing platform, we analyzed the dataset using linear-discriminant analysis (LDA), a supervised pattern-recognition algorithm that projects multivariate data onto a low-dimensional space where class separation is maximized. In the resulting LDA score plot, each point represents the fluorescence-response fingerprint of an individual sample measured by the sensor array (Fig. 2D and S4B). The first and second axes represent the linear-discriminant functions that provide maximum and second-maximum separation between classes, respectively. Notably, the clusters corresponding to each serum were clearly resolved without overlap. To quantify the classification performance, we performed two validation tests, leave-one-out cross-validation and holdout validation, both of which yielded 100% accuracy in serum identification (Dataset S1). These results demonstrate the highly effective nature of our hypothesis-free approach for the discrimination of complex serum compositions.

To further explore the relationships between the fluorescence response fingerprints, we performed principal-component analysis (PCA), an unsupervised dimensionality-reduction technique that projects data based on variance. Interestingly, in the score plot based on the first two principal components, we observed the emergence of meta-clusters corresponding to the taxonomic orders Artiodactyla and Rodentia (Fig. 2E and S4C). To more rigorously assess this feature-extraction capability, we re-labeled the serum samples according to their taxonomic order and subjected the dataset to LDA-based meta-analysis (Fig. S4D). The resulting plot showed high-resolution discrimination of the six taxonomic orders and even suggested the presence of separable trends at the superorder level. Next, we quantitatively examined the relationship between the fluorescence-response fingerprints and phylogenetic relatedness using a Mantel test comparing pairwise fluorescence-fingerprint distances (based on the Pearson correlation) with cytochrome *c* oxidase subunit I (COX1)-based genetic distances across 14 species. A statistically significant positive correlation was observed (Mantel *r* = 0.23, *p* < 0.01, 9999 permutations; Fig. S5), providing quantitative support for the taxonomic clustering patterns observed in the PCA and LDA analyses.

These findings suggest that the fluorescence-response fingerprints obtained from serum analysis partially reflect phylogenetic relationships, potentially arising from gradual, lineage-specific shifts in the molecular composition of the serum.

### Molecular basis of the fluorescence fingerprints

Proteins are a plausible major contributor to driving the observed discrimination, as due to their large molecular size, they strongly engage in multivalent interactions with the charged polymers. Among serum proteins, albumin is the most abundant and is therefore expected to substantially influence the sensor responses through nonspecific multivalent interactions. To directly examine this contribution, we performed albumin-depletion experiments on sera from six species using a commercial kit (AlbuSorb), which is validated for use with these species, and compared the fluorescence fingerprints before and after depletion. Albumin removal reduced fluorescence responses across most probes, with the magnitude varying considerably by probe and species, *i.e.*, moderate reductions were observed for most probes (20–40%), while certain probes showed a more pronounced decrease (*e.g.*, 50–70% for P-Suc and P-Pht) or minimal changes (*e.g.*, <10% for P-Pyr) (Fig. S6A and Dataset S2). These results indicate that albumin contributes to the overall fingerprint in a probe- and species-dependent manner, while they also demonstrate that other serum components make substantial contributions, as evident from the persistence of fluorescence responses after depletion.

An LDA revealed that albumin depletion altered the distribution of fingerprint patterns, including a redistribution of inter-group variance across discriminant axes (LDA score (1): 63.9% with depletion *vs.* 86.3% without depletion). However, species-specific clustering patterns, such as the proximity of mouse and rat, and of sheep and goat, were consistently observed regardless of depletion (Fig. S6B and C). Leave-one-out cross-validation accuracy was modestly lower after depletion (94% *vs.* 86%), which may in part reflect a decrease in signal intensity. Taken together, these results suggest that while albumin contributes to the fluorescence fingerprints in a probe- and species-dependent manner, the discriminative information embedded in the fingerprints is not exclusively attributable to albumin. Rather, the collective responses from multiple serum components appear to underlie the observed species discrimination.

Numerous other serum proteins differ in molecular size, charge, and hydrophobicity, and may therefore interact with the AIE-polymers in distinct ways. The collective responses arising from these interactions are likely integrated into complex fluorescence fingerprints that reflect characteristic features of the serum protein composition. Other classes of biomolecules may also influence the overall response fingerprints. Although small molecules such as amino acids, sugars, and vitamins are expected to interact more weakly with the polymers than proteins, their relatively high concentrations in serum may allow them to interact with the AIE-polymers according to their charge and hydrophobicity, thereby contributing measurably to the overall signal. These considerations collectively support the hypothesis-free, holistic nature of the present sensing platform.

### Reproducibility of the sensing platform

To evaluate the reproducibility of the sensor responses, two independent experimental batches were prepared, and each condition was measured in six replicate experiments (Dataset S3). The mean responses with standard deviations and the corresponding coefficients of variation (CVs) are summarized in Fig. S7. The CV values were generally below 10% for most sensor responses, indicating good reproducibility. Higher CV values were observed only for probes that exhibited very weak fluorescence responses (*e.g.*, P-Pht), where small absolute signal fluctuations resulted in relatively large relative variations. To further assess the robustness of the sensing patterns, fluorescence fingerprints from one experimental batch were used as the training dataset, whereas those from the other batch were used as the test dataset (Fig. S8). Assignment based on the Mahalanobis distance achieved an accuracy of 98% in this hold-out validation, demonstrating that the fluorescence fingerprints are highly reproducible across independent experimental batches.

Thus, our hypothesis-free polymeric sensing platform proved capable of capturing unexpected features embedded in the biochemical complexity of sera. In the following sections, we investigate whether this approach can be extended to address more practical challenges associated with the quality control of cell-culture supplements.

### Origin and lot identification

Differences in the origin and lots of serum often present a significant challenge to the reproducibility of cell-culture experiments and to the consistency and quality of cell-based products, primarily due to variations in composition that can affect cellular responses.^10^ To assess whether our hypothesis-free polymeric sensing platform could address this issue, we analyzed FBS samples from four countries of origin, with two distinct production lots from each origin.

**Fig. 3:**
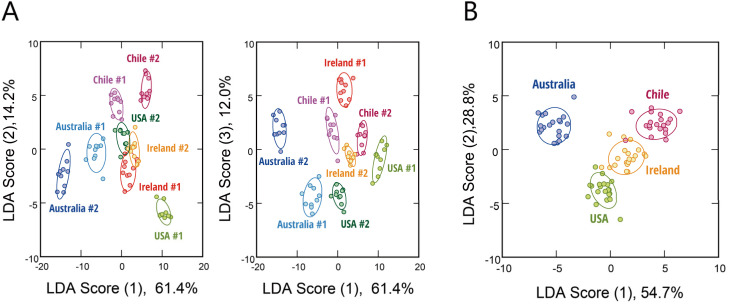
Optical identification of FBS origin and lot variation. (A and B) LDA score plots for the eight different FBS samples (0.10 vol%), wherein the analytes are labelled according to (A) both origin and lot and (B) origin only. (A), (left) score (1) *vs.* score (2); (A), (right) score (1) *vs.* score (3). For each analyte, ten independent experimental values are shown. The ellipsoids represent the confidence intervals (±1 SD) for each group.

The fluorescence-response fingerprints obtained using the six polymer probes (Fig. S9A and Dataset S4) were subjected to LDA. The first discriminant functions clearly separated all four origins and their respective lots (#1 and #2) without any overlap among clusters (Fig. 3A). The classification accuracy reached 99% in the leave-one-out cross-validation and 88% in the holdout test. Interestingly, samples clustered according to their geographic origin, and a meta-analysis using only the origin as the label demonstrated that the origin-specific classification was highly accurate (Fig. 3B). This indicates that differences between origins (potentially reflecting factors such as breed, feeding conditions, and manufacturing processes) are more pronounced than lot-to-lot variations (which may arise from factors such as donor pool composition or production timing). Notably, when these FBS samples were analyzed alongside sera from other animal species, all bovine sera were clustered tightly together and remained distinct from those of the other animals (Fig. S9B). This finding highlights that interspecies variation dominates over differences attributable to origin or lot, yet our system retains the sensitivity to detect both major and subtle differences in serum composition.

### Identification of storage conditions and heat-inactivation treatments

We next evaluated whether our platform could distinguish FBS samples subjected to different storage and processing conditions, an important aspect of serum quality control. To this end, we prepared samples from the same lot of FBS that had been stored under light-shielded and sealed conditions at 4 °C or 37 °C for up to four weeks. In addition, we prepared samples subjected to heating (56 °C for 30 or 60 minutes) to simulate typical heat-inactivation treatments.

**Fig. 4:**
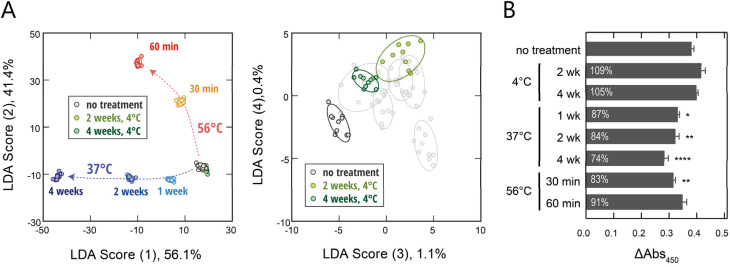
Optical identification of FBS samples held under different storage conditions and subjected to different heat-inactivation treatments. (A) LDA score plots for FBS (0.10 vol%) stored under the eight different conditions. (A), (left) score (1) *vs.* score (2); (A), (right) score (3) *vs.* score (4). For each analyte, ten independent experimental values are shown. The ellipsoids represent the confidence intervals (±1 SD) for each group. (B) Effect of FBS storage conditions on NHDF proliferation. NHDFs were cultured for 48 hours in Dulbecco's Modified Eagle Medium supplemented with 10 vol% of FBS stored under each condition. Intracellular dehydrogenase activity was subsequently assessed by measuring absorbance at 450 nm. Statistical analysis was performed using one-way ANOVA followed by Tukey's multiple comparison test (mean values ± 1 SE; *n* = 6). Asterisks indicate statistical significance compared to no treatment (*P < 0.05, **P < 0.01, ****P < 0.0001).

Fluorescence-response fingerprints for these samples were obtained using the six polymer probes (Fig. S10 and Dataset S5), and analyzed using LDA (Fig. 4A). The resulting score plots revealed that samples incubated at 37 °C and 56 °C formed distinct clusters that shifted along the first and second discriminant axes, respectively, whereas those stored at 4 °C showed separation along the third and fourth axes. These results demonstrate that our sensor array is capable of detecting temperature-dependent changes in serum composition with high classification accuracy (98% for leave-one-out cross-validation; 97% for a holdout test).

Importantly, most of the samples treated at 37 °C and 56 °C exhibited a significant reduction in the proliferation rate of normal human dermal fibroblasts (NHDFs) (Fig. 4B), confirming that the detected compositional changes correlate with functional deterioration. The 4 °C treatment had little impact on cell proliferation, consistent with the minimal changes observed in the fluorescence fingerprints. Notably, the actual effects of treatment on proliferation were relatively modest compared to the pronounced cluster shifts in the score plots. This indicates that our sensor array can sensitively detect subtle quality deterioration. For example, even treatment at 56 °C for 60 min resulted in only a slight decrease in proliferation rate. However, it remains unclear whether such changes would similarly affect other cellular functions or different cell types. This apparent discrepancy, where samples seem acceptable at first glance despite detectable compositional changes, is characteristic of cell-culture systems, highlighting the difficulty of evaluating quality solely by lot-check assays.

Taken together, the ability to distinguish both incubation temperature and treatment duration highlights the potential utility of this sensing approach as a rapid screening tool to assess whether a stored serum sample remains suitable for cell-culture use. Thus, our demonstrated approach serves as an alternative to conventional lot verification that requires time-intensive cell-based assays. In practical terms, this capability could help to improve yield and reproducibility in cell-culture workflows by enabling early identification and exclusion of lots with subtle but potentially detrimental quality changes before costly or large-scale experiments are initiated.

### Quality assessment of supplements for stem-cell culture

In the production of cell-based products for therapeutic purposes, such as regenerative medicine, the use of undefined components in animal-derived sera has raised considerable concerns. While chemically defined media (CDM) are preferred for such applications,^10^ and have achieved success for many cell types, complete serum replacement remains challenging in certain applications. Alternatively, partially defined media incorporating biological components offer a compromise approach. Regardless of the strategy used, most formulations require supplementation with proteins such as albumin, transferrin, growth factors, and hormones,^10,54^ which are often stress-sensitive and prone to denaturation or inactivation under suboptimal storage conditions. To address the challenge of supplement quality assessment, we applied our platform to evaluate a range of supplements used in stem-cell culture media and validated the approach by comparing sensor outputs to their effects on the proliferation and differentiation of pluripotent stem cells.

**Fig. 5:**
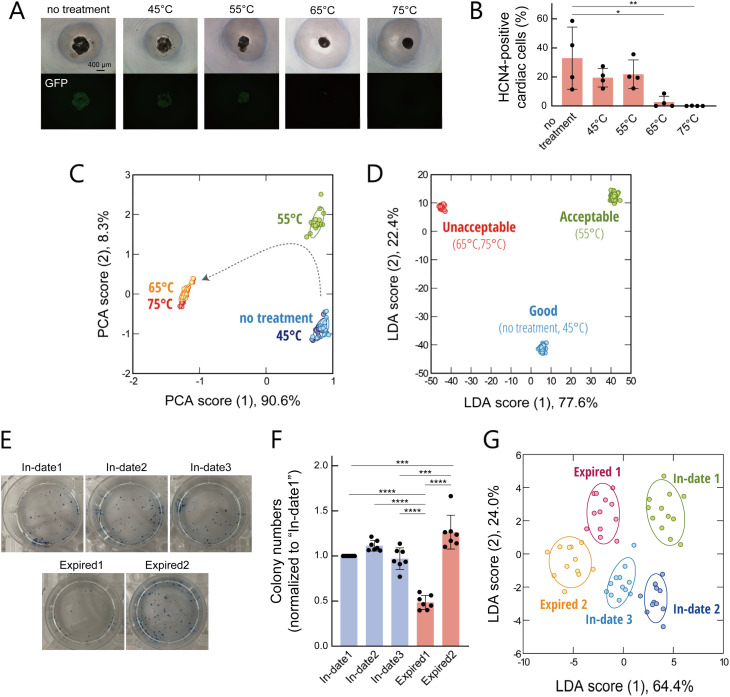
Optical assessment of serum-free supplements. (A) Bright-field and fluorescence-microscopy images and (B) the proportion of HCN4-positive cells measured by flow cytometry, following cardiomyocyte differentiation of HCN4-EGFP-hiPSCs using media supplemented with N2/B27 supplements processed at different temperatures. Statistical analysis was performed using one-way ANOVA followed by a Tukey's multiple comparison test (mean values ± 1 SE; *n* = 4). Asterisks indicate statistically significant differences between groups (*P < 0.05, **P < 0.01). (C) PCA score plot and (D) LDA score plot for cardiomyocyte-differentiation media (0.50 vol%) containing the processed N2/B27 supplements. Samples are labelled according to (C) processing conditions and (D) supplement quality. For each analyte, 18 independent experimental values are shown for each condition. The ellipsoids represent the confidence intervals (±1 SD) for each group. (E) Alkaline-phosphatase-staining images of hiPSC colonies cultured in media containing serum-free supplements stored for different durations, and (F) the corresponding number of positively stained colonies. Statistical analysis was performed using one-way ANOVA followed by a Tukey's multiple comparison test (mean values ± 1 SE; *n* = 7). Asterisks indicate statistically significant differences between groups (***P < 0.001, ****P < 0.0001). (G) LDA score plot for supplements (0.02 vol%) stored for different durations. For each analyte, 12 independent experimental values are shown. The ellipsoids represent the confidence intervals (±1 SD) for each group.

We first focused on the N2/B27 supplements, which are chemically defined supplements frequently used to support the maintenance, proliferation, and directed differentiation of stem cells.^55–57^ These supplements contain not only small molecules such as vitamins, but also proteins such as transferrin and insulin. To evaluate their thermal stability, the N2/B27 supplements were subjected to 30-minute treatments at various temperatures, and cardiomyocyte-differentiation media were prepared using the treated supplements. We employed a human-induced pluripotent stem-cell (hiPSC) line engineered to express enhanced green fluorescent protein (EGFP) under the control of the hyperpolarization-activated cyclic nucleotide-gated cation channel 4 (HCN-4) gene, a well-established marker of cardiac progenitors.^58^ In this reporter cell line, GFP expression is specifically induced in cells that have differentiated into cardiac progenitor cells.^59^ When these media were used to induce cardiomyocyte differentiation, no GFP-positive differentiated cells were observed using fluorescence microscopy when the supplements had been treated at 65 °C or higher (Fig. 5A). Flow-cytometric analysis quantitatively confirmed the same trend (Fig. 5B). These results demonstrate that thermal treatment impairs the capability of N2/B27 to induce differentiation in a temperature-dependent manner.

After confirming that the AIE-polymers P-None and P-Pht were able to respond to cardiomyocyte-differentiation media containing the N2/B27 supplements (Fig. S11A), we recorded the fluorescence fingerprints of these samples (Fig. S11B and Dataset S6) and analyzed them using PCA (Fig. 5C). The results showed that the cluster began to shift at 55 °C and moved further at 65 °C, but no additional changes were observed at higher temperatures. Based on the observed differentiation capacity and the fingerprint shifts detected by our system, we categorized the quality of each supplement as good, acceptable, or unacceptable and performed LDA. The resulting score plot revealed clearly separated clusters for each quality level without overlap (Fig. 5D), and both cross-validation tests yielded 100% accuracy in predicting the differentiation capability. These results demonstrate that our sensor array can detect subtle quality deterioration in supplements—even at levels that do not yet compromise cardiomyocyte-differentiation efficiency.

To assess a more practical application, we next evaluated a serum-free supplement used for maintaining the undifferentiated state of hiPSCs, which had been stored under appropriate freezing conditions. Specifically, we compared three unexpired supplements obtained within the manufacturer's specified shelf life (In-date1, In-date2, In-date3) and two expired supplements (Expired1, ∼4 years past expiry; Expired2, ∼12 years past expiry). The hiPSCs were cultured at a particular density for a fixed period in proliferation media prepared with each supplement. Colony and cell counts revealed that only the Expired1 condition led to a significant suppression of colony formation and cell growth (Fig. 5E, F and S12A, B).

These supplements were then analyzed using our sensor array (Fig. S12C, D and Dataset S7). LDA analysis with an optimized subset of the AIE-polymers not only successfully distinguished all five samples (with 97% accuracy in a leave-one-out test) but also revealed a clear boundary between the unexpired and expired groups (Fig. 5G). As with the results shown in Fig. 4, this finding indicates that our system can capture latent quality degradation not detectable through conventional proliferation assays. Such early detection could enable users to avoid using questionable media lots, thereby reducing the risk of compromised culture performance and supporting more reliable stem-cell-culture workflows.

### Recognition of naturally derived culture supplements

**Fig. 6:**
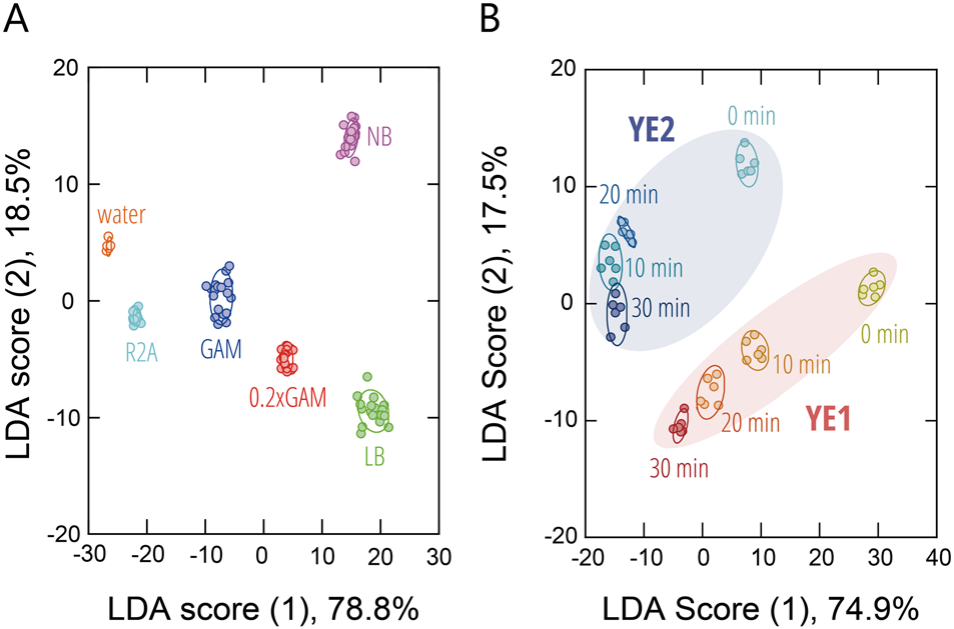
Optical identification of naturally derived culture supplements. LDA score plots for (A) microbial culture media (1.0 vol%) and (B) yeast extracts (1.0 mg mL^−1^) from two different manufacturers (YE1 and YE2), subjected to autoclave treatment for varying periods of time. For each analyte, 6 or 18 independent experimental values are shown. The ellipsoids represent the confidence intervals (±1 SD) for each group.

To examine the broader applicability of the sensing platform, we next extended our analysis to microbial culture media. Representative media commonly used for microbial cultivation (Gifu anaerobic medium (GAM), 0.2 × GAM, nutrient broth (NB), Luria–Bertani (LB) medium, and R2A broth) were analyzed. These media contain complex mixtures of naturally derived nutrients such as peptones, yeast extracts, and meat extracts, whose compositions vary depending on their intended applications. The resulting fluorescence fingerprints (Fig. S13 and Dataset S8) formed well-separated clusters in LDA (Fig. 6A), and leave-one-out cross-validation achieved a classification accuracy of 99%. These results demonstrate that the sensing platform can successfully discriminate complex microbial culture media composed of different natural ingredients.

Encouraged by these findings, we further investigated whether the system could detect differences in the state of yeast extracts, which are commonly used in microbial cultures central to the circular bioeconomy and in cost-sensitive applications such as cultured meat production. In microbial applications, it is standard practice to sterilize the media by autoclaving prior to use in order to prevent contamination. Taking this into account, we prepared yeast extracts from different manufacturers (YE1 and YE2) and subjected them to autoclave treatment at 121 °C for varying periods of time prior to analysis with our system. The resulting fluorescence fingerprints (Fig. S14 and Dataset S9) revealed that the yeast extracts from different manufacturers formed distinct clusters (Fig. 6B), with a classification accuracy of 96% in a leave-one-out test. Moreover, increasing autoclave duration led to a progressive shift in the clusters along the negative directions of the first and second linear-discriminant axes. These findings suggest that our system can be applied to rapidly evaluate batch-to-batch variation and differences in sterilization conditions for naturally derived supplements such as yeast extracts.

### Scope and limitations of the sensing platform

Finally, we discuss the scope and practical limitations of the present sensing platform. As demonstrated throughout this study, the hypothesis-free polymeric sensor platform successfully captured variations in a wide range of culture supplements, including animal sera, serum-free supplements used for stem-cell and microbial culture. These results suggest that the AIE-polymers interact with components of these supplements through multiple non-covalent interactions, including electrostatic, hydrophobic, and π–π interactions, thereby converting the overall compositional differences into fluorescence-response fingerprints.

Such cross-responsive sensing is particularly advantageous for naturally derived supplements, whose composition often varies depending on origin, lot, or manufacturing process. These variations reflect complex and often unpredictable changes in the overall molecular composition, which are well suited to detection by hypothesis-free sensing strategies based on non-specific interactions. Notably, the platform was also effective for partially defined media containing biological components. Compared with fully natural supplements, these media have relatively simpler and more controlled compositions. The observed discrimination in these cases likely arises from changes in labile components such as proteins that are sensitive to storage or processing conditions.

At the same time, the present results also provide insight into the practical detection limits of the current system. For example, only small differences were observed for FBS stored at 4 °C (Fig. 4A) and for frozen serum-free supplements (Fig. 5G), suggesting that the compositional changes under these conditions were close to the current detection threshold of the platform. In the present study, we employed hypothesis-free polymer probes without designing them for specific target molecules. Incorporating prior knowledge of components that are particularly sensitive to degradation, such as growth factors, cytokines, and metabolites, may enable further improvements in detection sensitivity through rational probe design.

## Conclusions

In this study, we developed and validated a hypothesis-free, data-driven polymeric sensing platform, constructed with an array of AIE-polymers, for the early and comprehensive quality assessment of complex culture media. The platform reliably distinguished a wide range of animal sera as well as various serum-free supplements used for stem-cell and microbial culture, capturing differences in type, origin, processing history, and quality status that are critical for reproducible cell manufacturing.

Beyond conventional quality assessment, the fluorescence-response fingerprints obtained from sera unexpectedly encoded phylogenetically informative signals, partially reflecting phylogenetic relationships among animal species. This finding indicates that our hypothesis-free polymeric sensor arrays can extract biologically meaningful information embedded in complex biological mixtures, extending their utility beyond predefined quality metrics and highlighting their potential as general analytical tools for comparative and systems-level studies in biotechnology.

Importantly, when applied to supplements used for culturing fibroblasts and pluripotent stem cells, the sensing platform detected not only overt quality deterioration that affected cell-culture outcomes, but also subtle compositional changes that had not yet impaired cell growth or differentiation. This capability for early detection suggests a practical route toward proactive quality assessment, in which potentially problematic media lots can be identified before costly or large-scale cell-culture experiments are initiated.

While the current implementation relies on standard microplate-based fluorescence measurements, the demonstrated robustness and reproducibility of this sensing strategy establish a foundation for broader adoption in cell manufacturing workflows. Future integration with more accessible or portable detection platforms could further expand its applicability. Overall, this work provides a generalizable and objective framework for evaluating complex culture media, with implications for improving standardization, reducing manufacturing variability, and supporting the reliable industrialization of cell-based technologies across biotechnology, regenerative medicine, and cellular agriculture.

## Author contributions

Conceptualization: S. T., K. M. and H. K.; data curation, formal analysis, investigation, methodology: S. T., K. M., N. K. and S. I.; funding acquisition: S. T.; resources: S. T., H. K., H. T. and R. K.; visualization, writing – original draft: S. T.; all authors discussed the results and contributed to the final manuscript.

## Conflicts of interest

The authors declare that they have filed patent applications related to this work.

## Supplementary Material

SC-OLF-D6SC00383D-s001

SC-OLF-D6SC00383D-s002

## Data Availability

The data supporting this article have been included as part of the supplementary information (SI). Supplementary information: experimental procedures, Fig. S1–S14 presenting absorption and fluorescence spectra, fluorescence-response heatmaps under various conditions, binding isotherms of AIE-polymers, microscopy images, and LDA and PCA score plots, as well as legends for the SI Datasets. See DOI: https://doi.org/10.1039/d6sc00383d.
