# Supplementary material for: A fingerprint-based polymeric sensing platform for comprehensive quality assessment of complex culture media in cell manufacturing
Source: Chem Sci. 2026 Apr 17;17(18):9006–17. doi: 10.1039/d6sc00383d (PMC13088908; doi:10.1039/d6sc00383d)
Supplement: SC-017-D6SC00383D-s002 [file SC-017-D6SC00383D-s002.pdf]

Supplementary Information (SI) for Chemical Science.
This journal is © The Royal Society of Chemistry 2026

Supplementary information for

A fingerprint-based polymeric sensing platform for comprehensive quality assessment of complex culture media in cell manufacturing

Shunsuke Tomita,[1,2] Kumi Morikawa,[3] Naoshi Kojima,[1] Sayaka Ishihara,[1] Hiroyuki Kusada[4], Hideyuki Tamaki,[4] and Ryoji Kurita*[1,5]

[1] Health and Medical Research Institute, National Institute of Advanced Industrial Science and Technology (AIST), 1-1-1 Higashi, Tsukuba, Ibaraki 305-8566, Japan

[2] School of Integrative and Global Majors, University of Tsukuba, Tsukuba, Ibaraki 305-8577, Japan

[3] Health and Medical Research Institute, AIST, 2217-14 Hayashi-cho, Takamatsu, Kagawa 761-0301, Japan

[4] Biomanufacturing Process Research Center, AIST, 1-1-1 Higashi, Tsukuba, Ibaraki 305-8566, Japan

[5] Faculty of Pure and Applied Sciences, University of Tsukuba, 1-1-1 Tennodai, Tsukuba, Ibaraki 305-8573, Japan

Materials and Methods

Materials

Pig serum, goat serum, guinea pig serum, horse serum, chicken serum, rabbit serum, mouse serum, sheep serum, rat serum, and human serum were obtained from Equitech-Bio Inc. Dog serum, cat serum, and hamster serum were obtained from Nordic-MUbio. Adult bovine serum, newborn calf serum, fetal bovine serum (FBS), 2-mercaptoethanol, Dulbecco's Modified Eagle Medium (DMEM)/F-12 (1:1 mixture), MEM non-essential amino acids, 3-morpholinopropanesulfonic acid (MOPS), nicotinamide, and dexamethasone were obtained from Sigma-Aldrich. FBS from different countries was obtained from Corning (U.S. and Australia) and Biosera (Ireland and Chile). KnockOut™ serum replacement (KSR), N-2 supplement, B-27 supplement without vitamin A, RPMI 1640 (without glutamine), penicillin-streptomycin, insulin-transferrin-selenium-G (ITS-G) supplement, and sodium pyruvate were obtained from Life Technologies. GlutaMAX™ Supplement and Geltrex were obtained from Thermo Fisher Scientific. DMEM (high glucose, with L-glutamine and phenol red), Y27632, L-ascorbic acid phosphate magnesium salt, and acetic acid were obtained from Wako Pure Chemical Industries. Recombinant human fibroblast growth factor (FGF) basic, recombinant human bone morphogenetic protein-4 (BMP-4), and human Noggin/Fc Chimera (carrier-free) were obtained from R&D Systems. CHIR99021 was obtained from Axon Medchem. IWP-2 was obtained from Cayman Chemical. Collagenase II was obtained from Worthington. AlbuSorb™ - Albumin Depletion Kit was obtained from Biotech Support Group LLC. Microbial culture media, including Gifu anaerobic medium (GAM) broth, nutrient broth (NB), Luria–Bertani (LB) medium (Miller formulation), and R2A broth, were obtained from Nissui, Biokar Diagnostics, Nacalai Tesque, and Nihon Pharmaceutical, respectively. A diluted medium (0.2×GAM) was prepared from GAM broth.

Methoxy-poly(ethylene glycol)-*block*-poly-L-lysine trifluoroacetate (PEG-*b*-PLL; degree of polymerization of PEG: 113; degree of polymerization of PLL: 52) was obtained from Alamanda Polymers, Inc. PEG-*b*-PLL modified with tetraphenylethylene (**P-None**), in which approximately 2.6 tetraphenylethylene moieties were conjugated to the primary amino groups of each PEG-*b*-PLL unit, was further fully modified at the remaining primary amino groups in the following ways according to literature procedures:[1] conjugation with amino acids bearing different aliphatic or aromatic groups (**P-Nle** or **P-Phe**, respectively); modification with acid anhydrides for cationic-to-anionic charge inversion (**P-Suc**, **P-Pht**, and **P-Pyr**).

Proliferation of fibroblasts using FBS after storage or heat treatment

Serum treatment. FBS was aliquoted into microtubes, sealed, protected from light, and subjected to one of the following storage or treatment conditions: 4 °C for 2 weeks or 4 weeks, 37 °C for 1 week, 2 weeks, or 4 weeks, and 56 °C for 30 minutes or 60 minutes. Untreated serum (immediately after thawing) was used as a control.

Evaluation of serum function using a fibroblast proliferation assay. Normal human dermal fibroblasts (NHDF; PromoCell GmbH) were suspended in DMEM at 4×10^4 cells/mL and seeded into 48-well plates (AGC Techno Glass) at 200 μL/well. Each FBS sample (treated or untreated) was added at 22.2 μL/well. After 48 h of incubation at 37 °C in a 5% CO_2 atmosphere, viable cell numbers were quantified using Cell Counting Kit-8 (Dojindo Laboratories) following the manufacturer's protocol.

Cardiomyocyte differentiation of human induced pluripotent stem cells (hiPSCs) using N2/B27 supplement after heat treatment

Heat treatment of supplements. The N-2 supplement (hereafter "N2") and B-27 supplement (hereafter "B27") were aliquoted into microtubes, sealed, protected from light, and subjected to heat stress at 45 °C, 55 °C, 65 °C, or 75 °C for 30 minutes. Control samples were left untreated. Following treatment, chemically defined medium 1 (CDM1), consisting of DMEM/F-12 supplemented with 1.0% N2, 2.0% B27, 1.0% MEM non-essential amino acids, 1.0% GlutaMAX™, 1.0% penicillin-streptomycin, and 0.1 mM 2-mercaptoethanol (referred to in the main text as cardiomyocyte differentiation media containing N2/B27 supplements) was prepared using either untreated or heat-treated N2 and B27.

Cardiomyocyte differentiation from hiPSCs. The hyperpolarization-activated cyclic nucleotide-gated cation channel 4 (HCN-4)-enhanced green fluorescent protein (EGFP) hiPSC line (409B2) provided by Dr. Shirayoshi Yasusaki (Graduate School of Medical Science, Tottori University), in which an EGFP cassette was knocked into the HCN4 gene locus via a BAC vector,[2] was suspended in mouse embryonic fibroblast-conditioned medium (MEF-CM) and seeded onto culture plates coated with Geltrex. On the following day, the medium was replaced with CDM1 supplemented with 100 ng/mL Noggin and 3.3 µM CHIR99021. The medium was refreshed daily with fresh CDM1 until day 3. On day 4, the medium was replaced with CDM1 supplemented with 3.3 µM CHIR99021 and 5.0 µM IWP-2. On day 5, cells were dissociated using trypsin and re-seeded at a density of 1×10^4 cells/well into PrimeSurface™ 96U low-attachment plates in cardiomyocyte differentiation medium 2 (CDM2), consisting of RPMI 1640 supplemented with 1% sodium pyruvate, 1% GlutaMAX™, 1.0% ITS-G supplement, 2.0 mM L-ascorbic acid, 10 mM nicotinamide, 0.20 µM dexamethasone, 0.50% FBS, 1.0% penicillin-streptomycin, 10 ng/mL recombinant human bFGF, and 10 ng/mL recombinant human BMP-4. The medium was refreshed every 2–3 days.

Assessment of differentiation efficiency. On day 19 or 20, GFP fluorescence was observed using a fluorescence microscope (Olympus IX73). For flow cytometric analysis, cells were treated with 2 mg/mL collagenase II and incubated overnight at room temperature. The resulting single-cell suspension was analyzed using a FACSAria™ III cell sorter (BD Biosciences), and the percentage of GFP-positive (i.e., HCN4-expressing) cells was quantified.

Maintenance and proliferation of hiPSCs using KSR after storage

Preparation of culture media using KSR with different storage periods. KSR samples stored at –80 °C were used, including three within the manufacturer's expiration period (In-date1, In-date2, In-date3) and two expired samples (Expired1: ~4 years past expiration; Expired2: ~12 years past expiration). The proliferation medium was prepared by supplementing DMEM/F-12 with 20% KSR, 1.0% MEM non-essential amino acids, 1.0% GlutaMAX™, 1.0% penicillin-streptomycin, and 0.1 mM 2-mercaptoethanol.

Maintenance of human hiPSCs and alkaline phosphatase staining. The 409B2 hiPSC line was suspended in the proliferation medium containing 5.0 ng/mL bFGF and 10 µM Y-27632. Cells were seeded at a density of 1×10^4 cells/well onto 6-well plates (Corning) coated with Matrigel (Corning). The culture medium was replaced every three days with fresh proliferation medium supplemented with 5.0 ng/mL bFGF. On day 9 post-seeding, alkaline phosphatase (AP) staining was performed using the AP staining kit (System Biosciences), and the number of positively stained undifferentiated colonies was counted.

Measurement of cell proliferation. For cell number quantification, hiPSCs were seeded at a density of 1×10^5 cells/well under the same culture conditions. The medium was exchanged daily with fresh proliferation medium containing 5.0 ng/mL bFGF. On day 4 post-seeding, microscopy images were captured, followed by trypsinization and recovery of single-cell suspensions. Cell numbers were counted using a hemocytometer.

Fluorescence measurements of the polymers

Preliminary characterization. Solutions (300 μL) containing the aggregation-induced emission (AIE)-polymers (10.0 μM) in 10.0 mM HCl or 10.0 mM NaOH were placed in 1 cm pathlength quartz cuvette for absorption measurements or in each well of a 384-well NBS™ black microplate (Corning) for fluorescence measurements. Absorption spectrum was recorded at room temperature using a V-750 spectrophotometer (JASCO). Fluorescence spectrum ($\lambda_{ex}/\lambda_{em}$ = 330 nm/372–700 nm) or the fluorescence intensity ($\lambda_{ex}/\lambda_{em}$ = 330 nm/480 nm) were recorded at 35 °C.

Serum. Solutions (180 μL) containing the AIE-polymers (333 nM) in 20.0 mM MOPS buffer (pH = 7.0) were prepared in each well of a 96-well NBS™ black microplate (Corning) using an Andrew+ pipetting robot (Andrew Alliance). After incubation (35 °C, 10 min), the fluorescence spectrum ($\lambda_{ex}/\lambda_{em}$ = 330 nm/372–700 nm) or the fluorescence intensity ($\lambda_{ex}/\lambda_{em}$ = 330 nm/480 nm) were recorded at 35 °C using a Cytation5 Imaging Reader (BioTek). Subsequently, aliquots (20 μL) containing various concentrations of human serum in 20.0 mM MOPS buffer (pH = 7.0) were added to each well, and the fluorescence spectrum or intensity was recorded after incubation (35 °C, 10 min). The final concentrations were 300 nM AIE-polymer, 0–0.500 vol% serum, in 20.0 mM MOPS buffer (pH = 7.0).

Medium for stem cell culture. Solutions (45 μL) containing 400 nM AIE-polymer in 20.0 mM MOPS buffer (pH = 7.0) were prepared in each well of a 384-well NBS™ black microplate (Corning) using an Andrew+ pipetting robot. After incubation (35 °C, 10 min), the fluorescence intensity ($\lambda_{ex}/\lambda_{em}$ = 330 nm/480 nm) was recorded at 35 °C using a Cytation5 Imaging Reader. Subsequently, aliquots (15 μL) containing various concentrations of CDM1 or KSR were added to each well, and the fluorescence intensity was recorded after incubation (35 °C, 10 min). The final concentrations were 300 nM AIE-polymer, 0–2.0 vol% CDM1 or 0–0.1 vol% KSR, in 20.0 mM MOPS buffer (pH = 7.0).

Hypothesis-free polymeric sensing

Fluorescence responses of serum. Aliquots (108 μL) of solutions containing the AIE-polymers (333 nM) in 22.2 mM MOPS buffer (pH = 7.0) or 22.2 mM acetate buffer (pH = 5.0) were deposited in the wells of a 96-half-well NBS™ black microplate (Corning) using an Andrew+ pipetting robot. After incubation (35 °C, 10 min), the fluorescence intensity was recorded using two different channels (Ch1: $\lambda_{ex}/\lambda_{em}$ = 330 nm/480 nm; Ch2: $\lambda_{ex}/\lambda_{em}$ = 360 nm/530 nm) using a Cytation5 Imaging Reader. Subsequently, aliquots (12 μL) of serum diluted in distilled water were added to each well, and the fluorescence intensity was recorded after incubation (35 °C, 10 min). The final concentrations were 300 nM AIE-polymer, 0.10 vol% serum, in 20.0 mM buffer. This process was performed 10 times for individual samples to generate a training data matrix. Albumin depletion experiments were performed according to the supplier-provided protocol using 25 μL of serum, and the eluted albumin-depleted serum was used for the fluorescence measurements.

Fluorescence responses of the stem cell culture medium. Aliquots (50 μL) of solutions containing the AIE-polymers (360 nM) in 24.0 mM MOPS buffer (pH = 7.0) or 24.0 mM acetate buffer (pH = 5.0) were deposited in the wells of a 384-well

NBS™ black microplate using an Andrew+ pipetting robot. After incubation (35 °C, 10 min), the fluorescence intensity was recorded using two different channels (Ch1: $\lambda_{ex}/\lambda_{em}$ = 330 nm/480 nm; Ch2: $\lambda_{ex}/\lambda_{em}$ = 360 nm/530 nm) using a Cytation5 Imaging Reader. Subsequently, aliquots (10 µL) of 3.0 vol% CDM1 diluted in distilled water or 0.12 vol% KSR diluted in distilled water were added to each well, and the fluorescence intensity was recorded after incubation (35 °C, 10 min). The final concentrations were 300 nM AIE-polymer, 0.5 vol% CDM1 or 0.02 vol% KSR, in 20.0 mM buffer. This process was performed at least 12 times for individual samples to generate a training data matrix. For the KSR analysis, the average of two measurements was used to improve reproducibility. Based on their contribution to classification performance, the combinations shown in Table S1 were selected for analysis.

Table S1. Variables used in the analysis of KSR.

Polymer	pH	Channel	Variables used
**P-None**	pH7.0	Ch1	✓
		Ch2	✓
	pH5.0	Ch1	✓
		Ch2	✓
**P-Nle**	pH7.0	Ch1	✓
		Ch2	✓
	pH5.0	Ch1	
		Ch2	
**P-Phe**	pH7.0	Ch1	✓
		Ch2	
	pH5.0	Ch1	✓
		Ch2	✓
**P-Suc**	pH7.0	Ch1	✓
		Ch2	
	pH5.0	Ch1	✓
		Ch2	
**P-Pht**	pH7.0	Ch1	
		Ch2	
	pH5.0	Ch1	
		Ch2	✓
**P-Pyr**	pH7.0	Ch1	✓
		Ch2	✓
	pH5.0	Ch1	✓
		Ch2	✓

Fluorescence responses of naturally derived culture supplements. For yeast extracts, powdered yeast extracts from two different manufacturers (Becton Dickinson and Oxoid, referred to as YE1 and YE2, respectively) were dissolved in distilled water at a concentration of 10.0 mg/mL, and 10 mL of each solution was transferred into a 15 mL centrifuge tube. The solutions were autoclaved at 121 °C for 10–30 min. Aliquots (50 µL) of solutions containing microbial culture media (1.2 vol%) or yeast extracts (1.2 mg/mL) diluted in distilled water in 24.0 mM MOPS buffer (pH = 7.0) or 24.0 mM acetate buffer (pH = 5.0) were deposited in the wells of a 384-well NBS™ black microplate using an ASSIST PLUS pipetting robot (Integra Biosciences). After incubation (35 °C, 10 min), the fluorescence intensity was recorded using two different channels (Ch1: $\lambda_{ex}/\lambda_{em}$ = 330 nm/480 nm; Ch2: $\lambda_{ex}/\lambda_{em}$ = 360 nm/530 nm) using a Synergy H1. Subsequently, aliquots (10 µL) of the AIE-polymers (900 nM with microbial culture media or 1800 nM with yeast extracts) in distilled water were added to each well, and the

fluorescence intensity was recorded after incubation (35 °C, 10 min). The final concentrations were 150 nM TPE-polymer, 1.0 vol% microbial culture media in 20.0 mM buffer, or 300 nM AIE-polymer, 1.0 mg/mL yeast extract in 20.0 mM buffer. This process was performed at least six times for the individual samples to generate a training data matrix.

Statistical analysis. The training data matrix was processed using linear discriminant analysis (LDA), hierarchical clustering analysis (HCA), and principal component analysis (PCA) in SYSTAT 13 (Systat Inc.). For cross-validation testing, a portion of the fluorescence fingerprints for each analyte was separated from the training data matrix and used as a test data matrix. The test data were classified into groups generated by the remaining training matrix according to their shortest Mahalanobis distances. HCA dendrograms were created based on the Euclidean distances using the Ward method and a dataset standardized prior to analysis using the following equation: $z = (x - \mu)/\sigma$, where z is the standardized score, x is the raw score, μ is the mean of the population, and σ is the standard deviation of the population. To quantitatively assess the phylogenetic encoding of the fluorescence fingerprints, a Mantel test was performed using pairwise-distance matrices for 14 animal species. Fluorescence-fingerprint distances were calculated as correlation distances ($1 - \text{Pearson } r$) from the z-score-standardized 24-dimensional response vectors. Phylogenetic distances were calculated as p-distances from the multiple sequence alignment of cytochrome *c* oxidase subunit I (COX1) gene sequences obtained from NCBI RefSeq database. Sequences were aligned using MAFFT (v7, web server). The Mantel test was performed using the mantel function in scikit-bio (v0.7.2; Pearson method, 9,999 permutations).

Figures

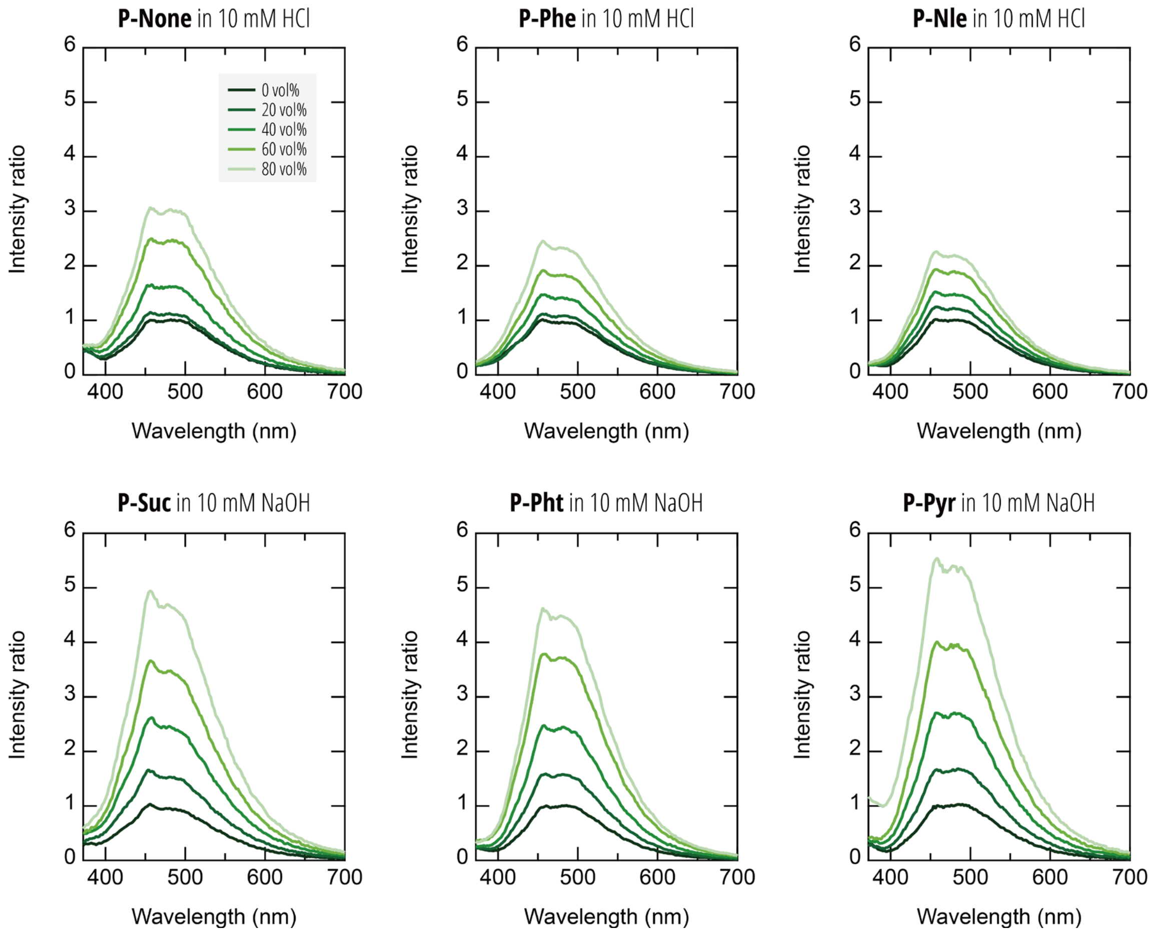

Fig. S1. Fluorescence spectra of AIE-polymers (2.0 μM) with increasing glycerol concentration (0–80 vol%) in 10 mM HCl for the cationic polymers (**P-None**, **P-Phe**, and **P-Nle**) or 10 mM NaOH for the anionic polymers (**P-Suc**, **P-Pht**, **P-Pyr**); λ_{ex} = 330 nm.

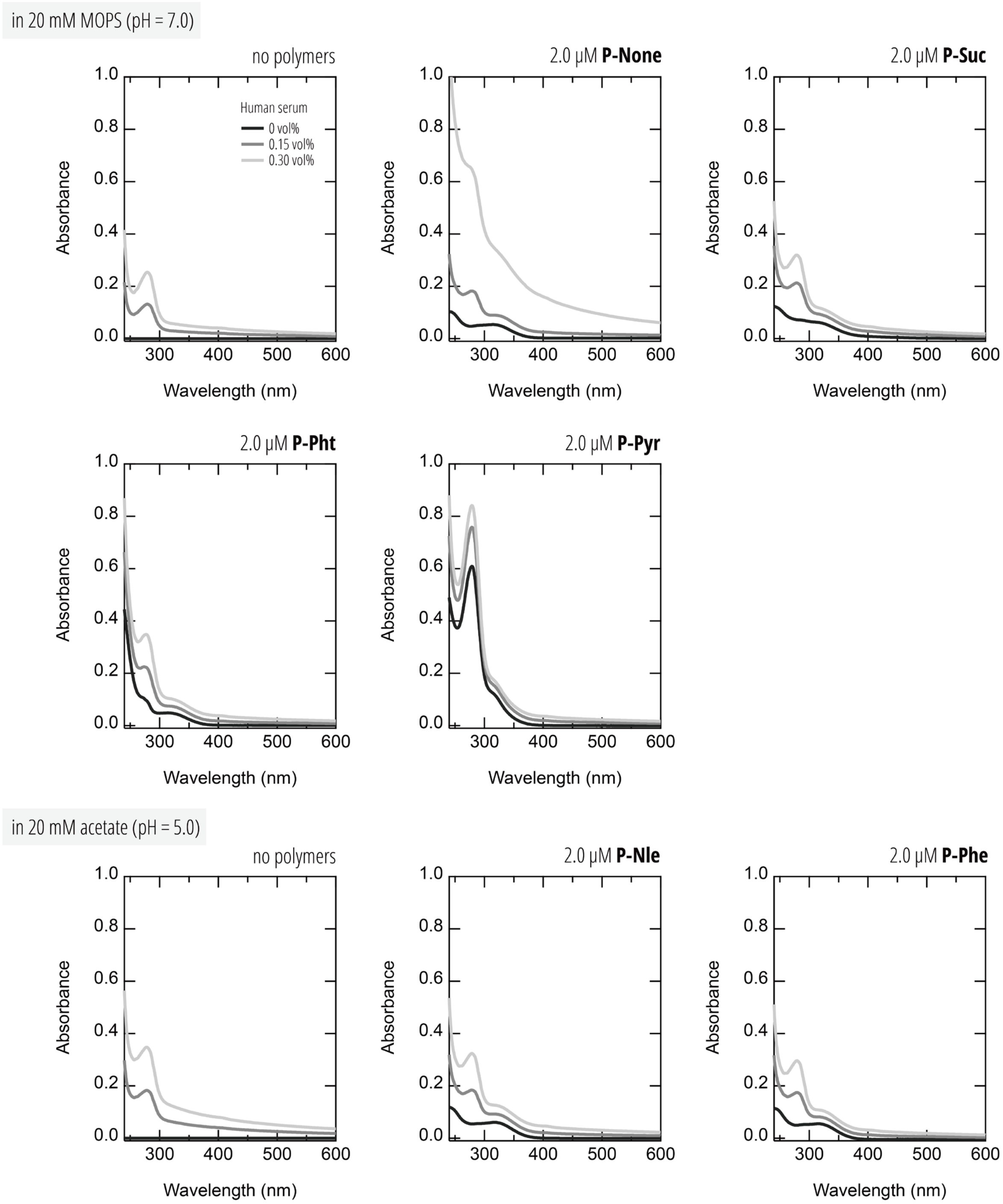

Fig. S2. Absorption spectra of AIE-polymers (2.0 μM) before and after addition of human serum (0–0.30 vol%) in 20 mM MOPS (pH = 7.0) for **P-None**, **P-Suc**, **P-Pht**, and **P-Pyr** or 20 mM acetate (pH = 5.0) for **P-Phe** and **P-Nle**. For **P-Phe** and **P-Nle**, the measurements were performed at pH = 5.0 to minimize self-induced fluorescence enhancement observed at pH = 7.0. Characteristic absorption bands attributable to the TPE moieties were observed for all polymers, with a prominent band around 315 nm.

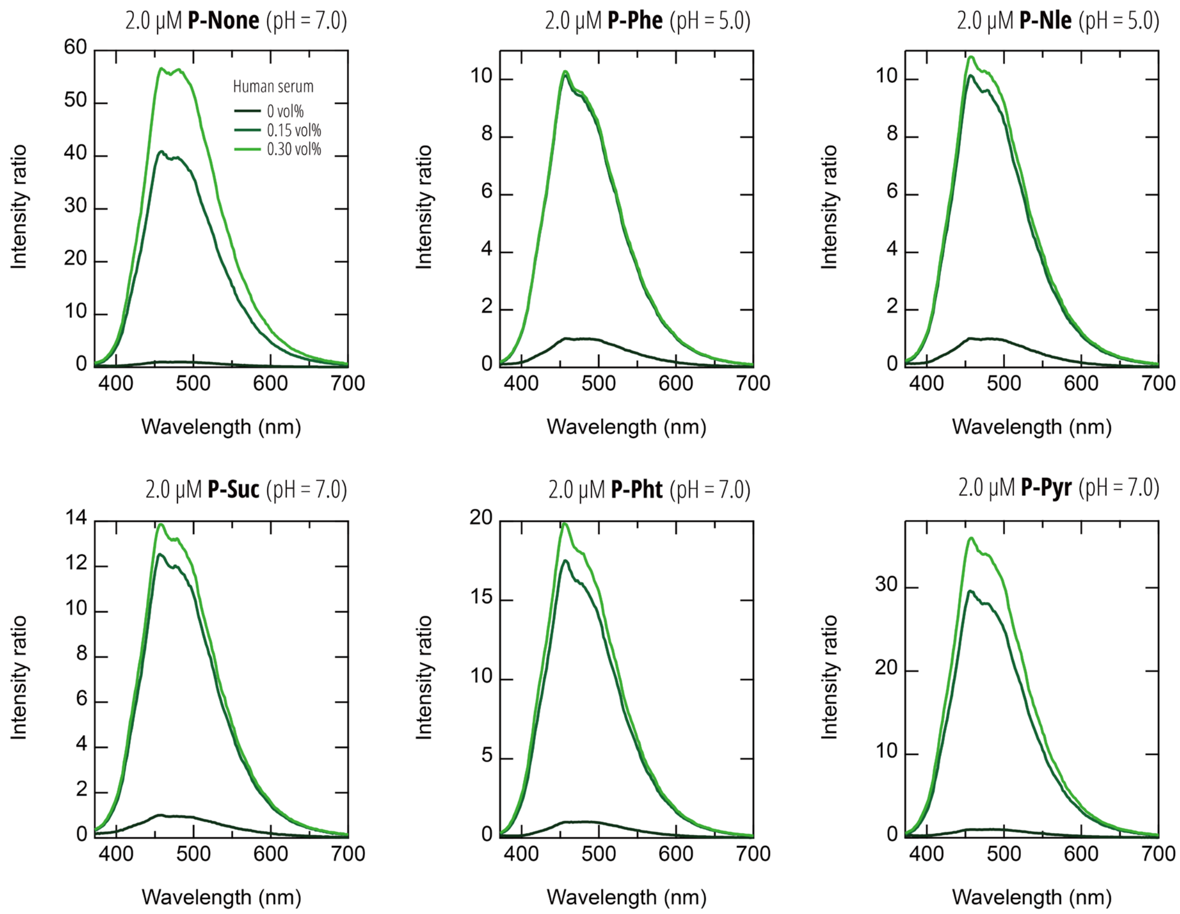

Fig. S3. Fluorescence spectra of AIE-polymers (2.0 μM) before and after addition of human serum (0–0.30 vol%) in 20 mM MOPS (pH = 7.0) for **P-None**, **P-Suc**, **P-Pht**, and **P-Pyr** or 20 mM acetate (pH = 5.0) for **P-Phe**, and **P-Nle**; λ_{ex} = 330 nm. For **P-Phe** and **P-Nle**, the measurements were performed at pH 5.0 to minimize self-induced fluorescence enhancement observed at pH = 7.0.

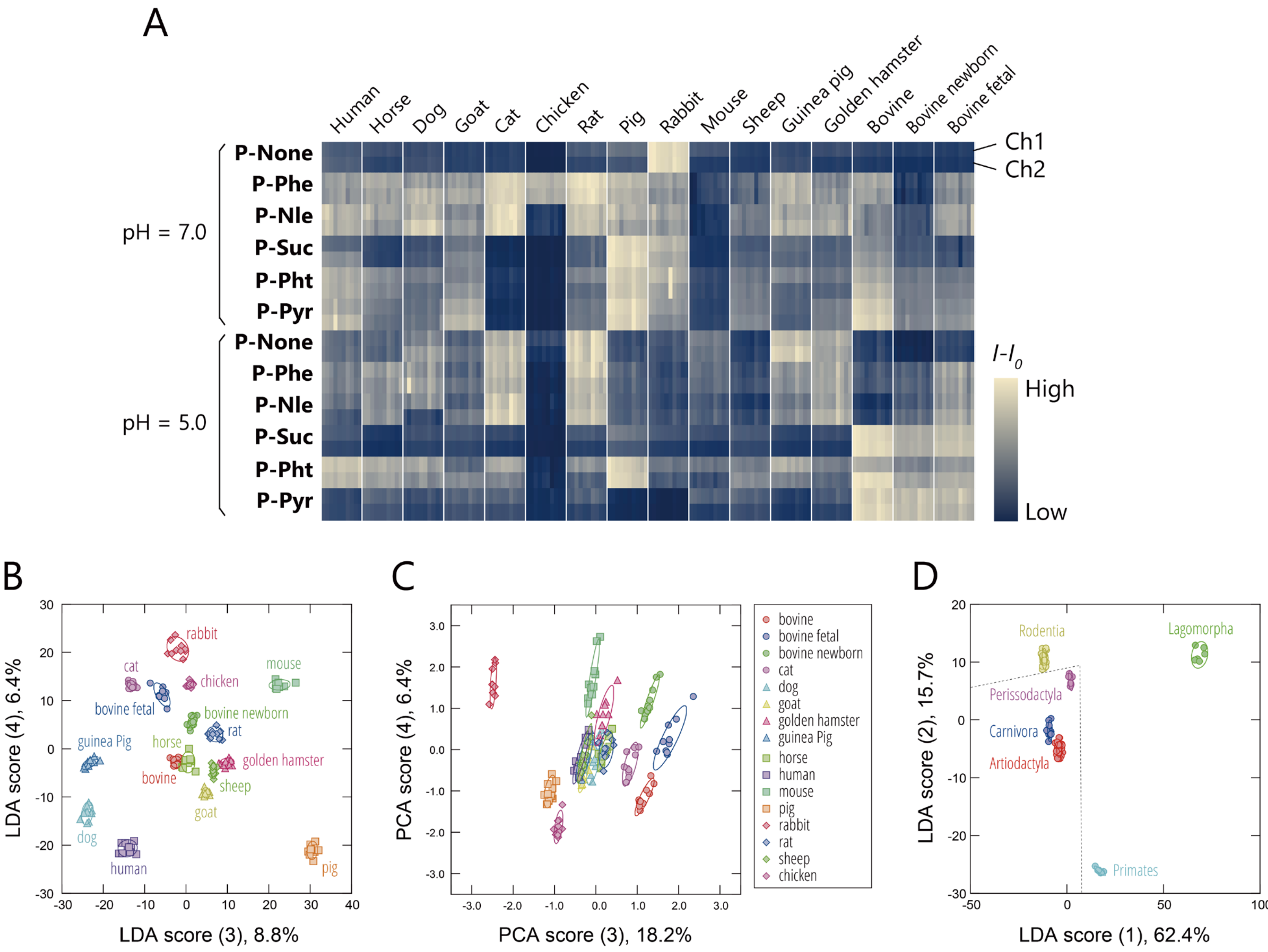

Fig. S4. (A) Heatmap of the fluorescence-response fingerprints of the 16 different sera (0.10 vol%). For each analyte, 10 independent experimental values are shown. (B, D) LDA score plots and (C) PCA score plot for the sera, wherein the analytes are labelled according to (B, C) the species and (D) the orders. (B, C) Score (3) vs. score (4); (D) score (1) vs. score (2). Ellipsoids represent the confidence intervals (±1 SD) for each analyte. In (D), the dashed line indicates the boundary between the two superorders, *Laurasiatheria* and *Euarchontoglires*.

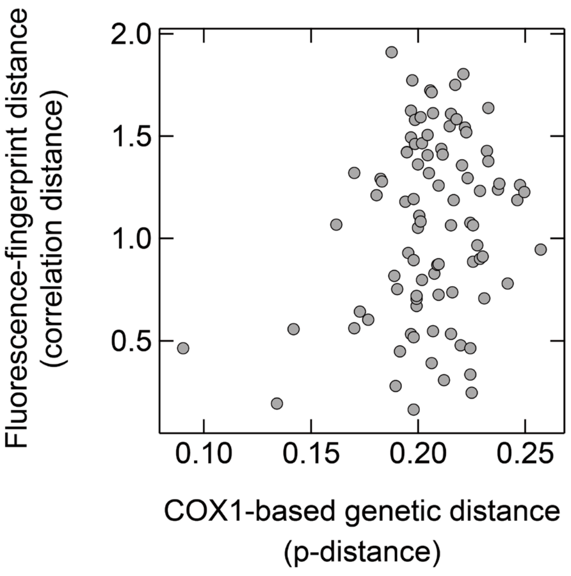

Fig. S5. Correlation between the fluorescence-fingerprint distance and the phylogenetic distance. Each point represents one of the 91 pairwise comparisons among 14 animal species. The *x*-axis shows the COX1-based genetic distance (p-distance), and the *y*-axis shows the fluorescence fingerprint distance (correlation distance, 1 − Pearson *r*). Mantel $r = 0.23$, $p < 0.01$ (Pearson Mantel test, Pearson method, 9,999 permutations).

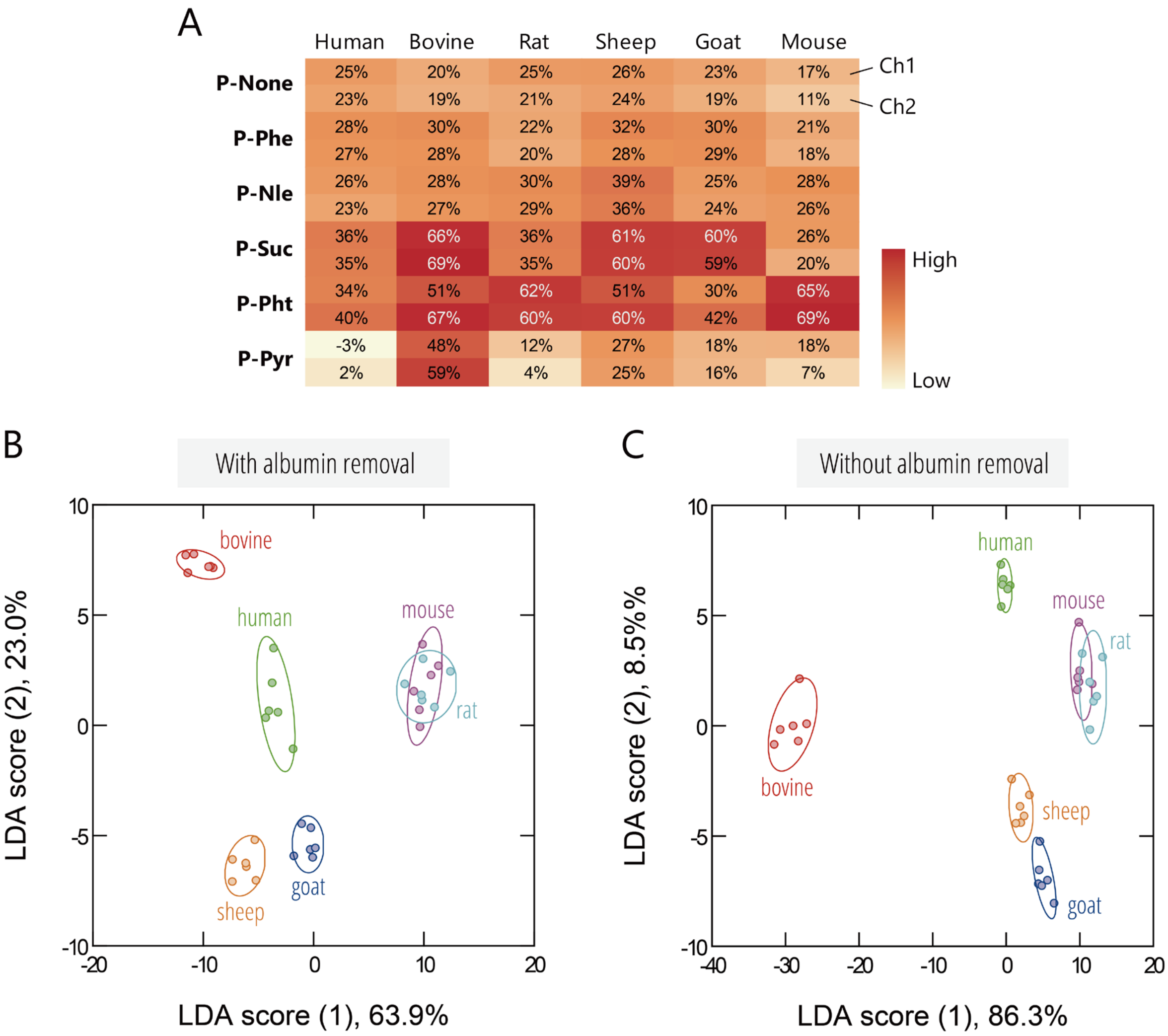

Fig. S6. Effect of albumin depletion on fluorescence-response fingerprints. (A) Relative contribution of albumin to the fluorescence response of each probe–channel combination, calculated as (non-depleted − depleted) / non-depleted × 100 (%). Higher values indicate greater albumin contribution to the corresponding response. (B, C) LDA score plots for the six serum samples (0.10 vol%) with (B) or without (C) albumin depletion. For each species, six independent measurements are shown. Ellipses represent the confidence intervals (±1 SD) for each group.

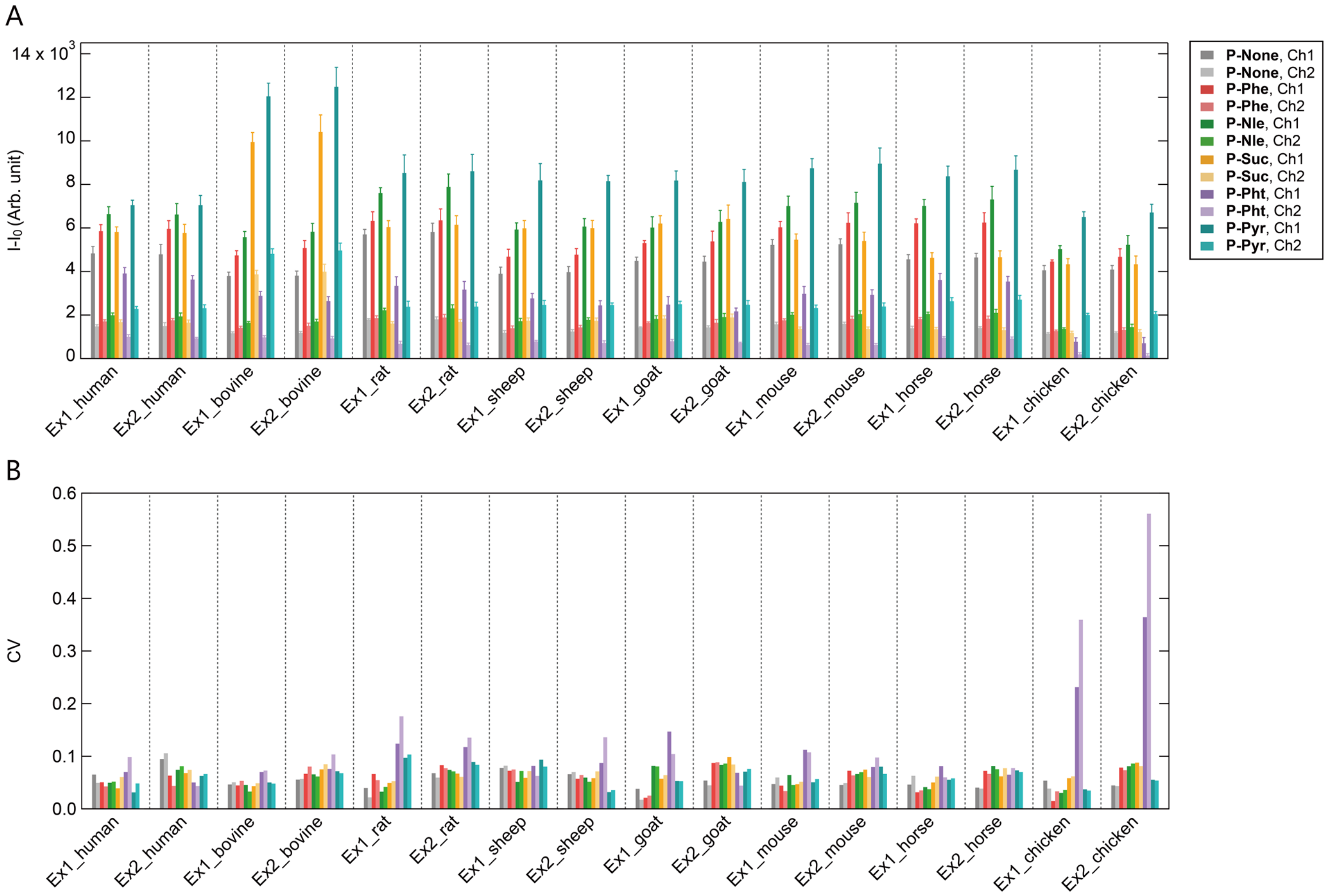

Fig. S7. Reproducibility of the fluorescence responses across two independent experimental batches (Ex1 and Ex2) for animal serum samples (0.10 vol%) measured using the array of six AIE-polymers in 20 mM acetate (pH = 5.0). (A) Fluorescence-intensity changes for each species in the two independent experiments. Error bars represent standard deviations (n = 6). (B) Coefficients of variation (CVs) calculated from the data shown in (A). The CV values were generally below 10% for most sensor responses, indicating good reproducibility. Higher CV values were observed only for probes that exhibited very weak fluorescence responses (e.g., **P-Pht**), where small absolute signal fluctuations resulted in relatively large relative variations.

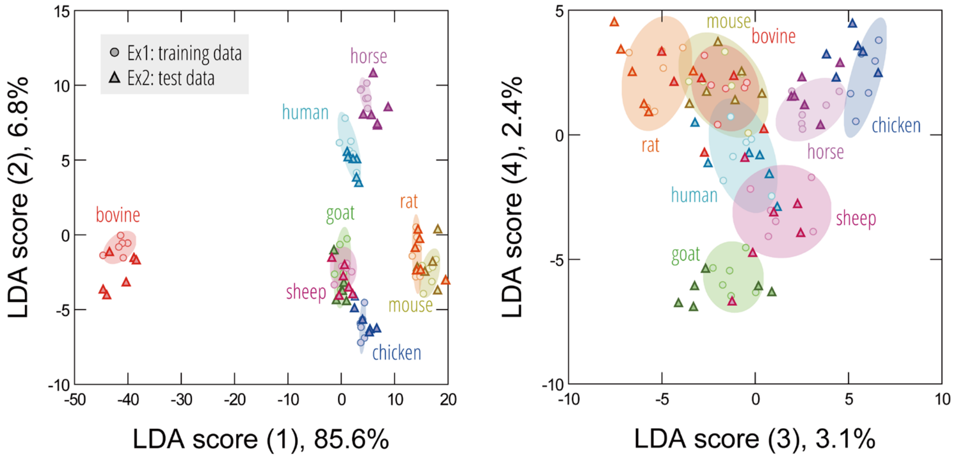

Fig. S8. Inter-batch reproducibility of the LDA classification for animal serum samples (0.10 vol%) measured using the array consisting of six AIE-polymers in 20 mM acetate (pH = 5.0). The discriminant functions were derived from the Ex1 training dataset (circles) and subsequently applied to the Ex2 test dataset (triangles). (Left) score (1) vs. score (2); (Right) score (3) vs. score (4). For each species, six independent measurements are shown. Ellipses represent ±1 SD for each group.

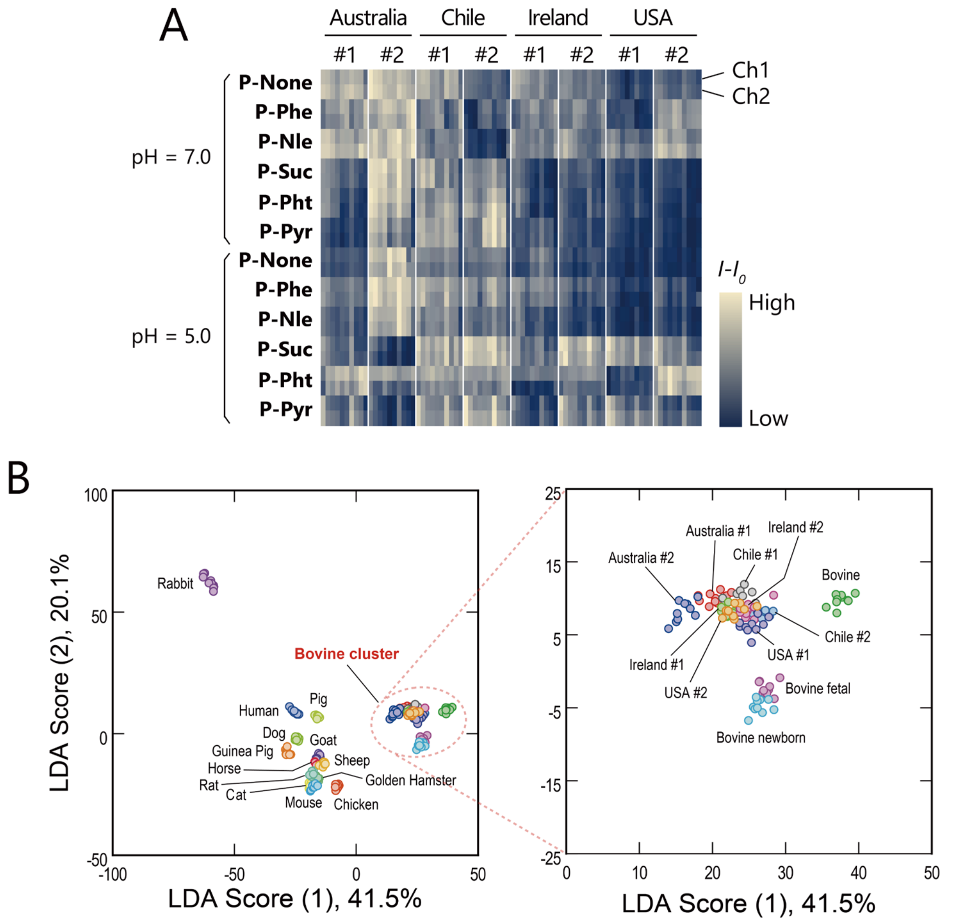

Fig. S9. (A) Heatmap of the fluorescence-response fingerprints of the 16 different FBS (0.10 vol%) samples. For each analyte, 10 independent experimental values are shown. (B) LDA score plots for the sera, obtained by combining the datasets shown in Figs. S4 and S9.

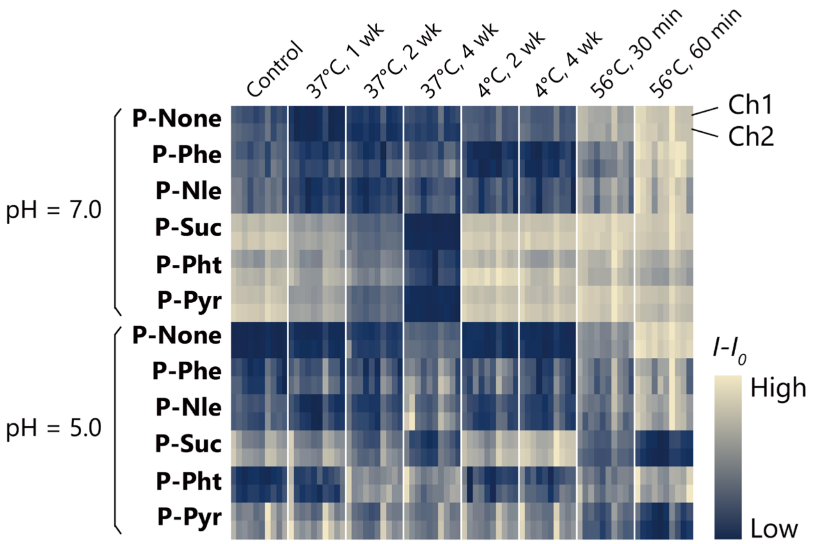

Fig. S10. Heatmap of the fluorescence-response fingerprints of FBS (0.10 vol%) stored under eight different conditions. For each analyte, 10 independent experimental values are shown.

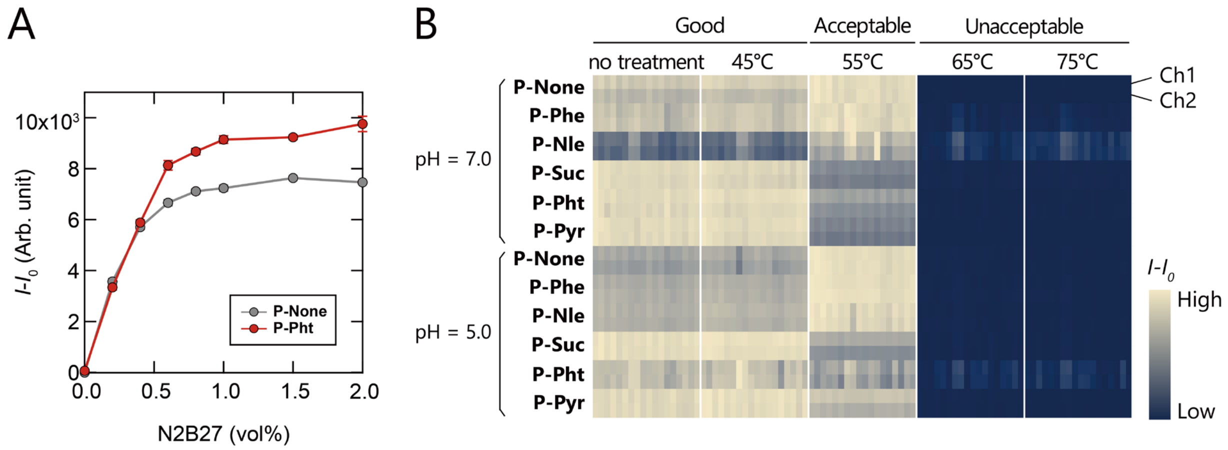

Fig. S11. (A) Binding isotherms for different AIE-polymers (300 nM) upon addition of cardiomyocyte differentiation media containing the N2/B27 supplements in 20 mM MOPS buffer (pH = 7.0); $\lambda_{ex}/\lambda_{em}$ = 330 nm/480 nm. The values shown represent mean values ±1 SE from three independent experiments. (B) Heatmap of the fluorescence-response fingerprints of cardiomyocyte differentiation media containing the N2/B27 supplements (0.50 vol%) treated under five different conditions. For each analyte, 18 independent experimental values are shown.

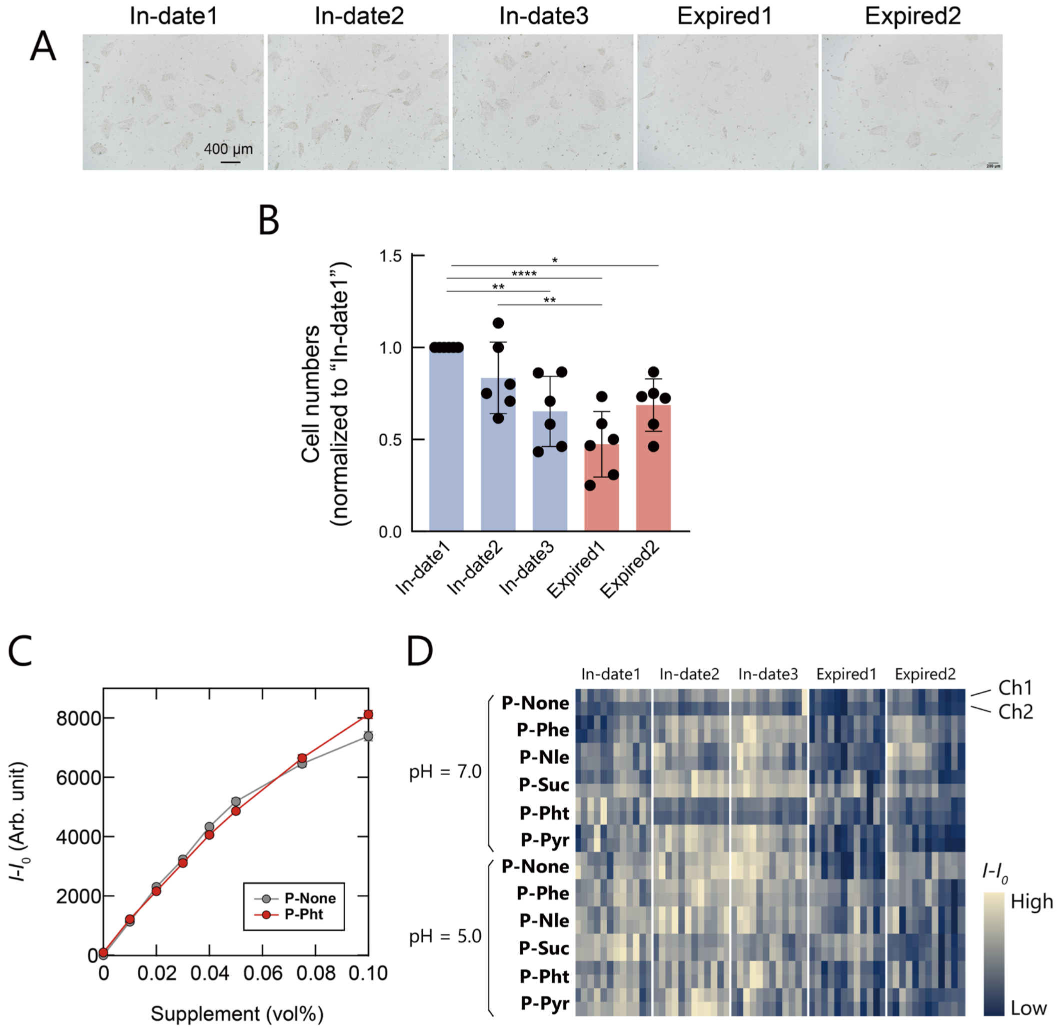

Fig. S12. (A) Bright-field microscopy images and (B) cell numbers of hiPSCs cultured in media containing serum-free supplements stored for different durations. Statistical analysis was performed with one-way ANOVA followed by a Tukey's multiple comparison test (mean values ±1 SE; n = 6). Asterisks indicate statistically significant differences between groups (*$P < 0.05$, **$P < 0.01$, ****$P < 0.0001$). (C) Binding isotherms for different AIE-polymers (300 nM) upon addition of serum-free supplements stored for different periods, measured in 20 mM MOPS buffer (pH = 7.0); $\lambda_{ex}/\lambda_{em}$ = 330 nm/480 nm. The values shown represent mean values ±1 SE from three independent experiments. (D) Heatmap of the fluorescence-response fingerprints of supplements (0.02 vol%) stored for different periods. For each analyte, 12 independent experimental values are shown.

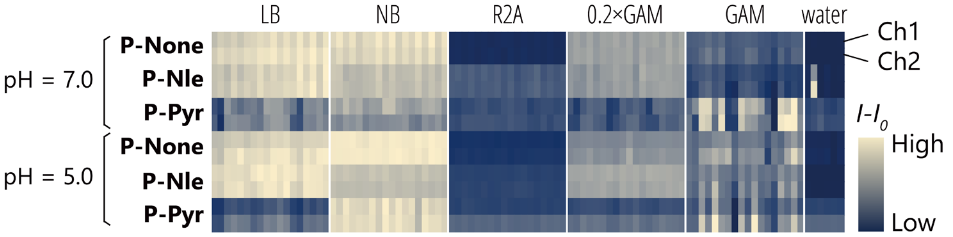

Fig. S13. Heatmap of the fluorescence-response fingerprints of microbial culture media (1.0 vol%). For each analyte, 6 or 18 independent experimental values are shown.

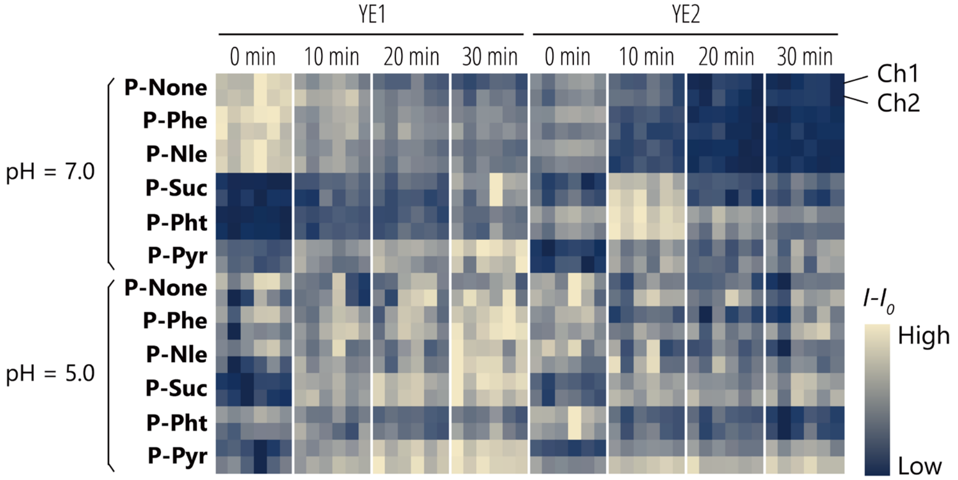

Fig. S14. Heatmap of the fluorescence-response fingerprints of yeast extracts (1.0 mg/mL) from two different manufacturers, subjected to autoclave treatment for four different durations. For each analyte, 6 independent experimental values are shown.

Dataset S1 (separate file). Data set matrix of the differences in fluorescence intensity before and after the addition of animal sera (0.10 vol%) to the AIE-polymers. The rightmost column indicates whether the data in the corresponding row was used as training data (denoted by "–") or test data (results of the verification are shown) in the holdout test.

Dataset S2 (separate file). Data set matrix of the differences in fluorescence intensity before and after the addition of albumin-depleted or non-depleted animal sera (0.10 vol%) to the AIE-polymers.

Dataset S3 (separate file). Data set matrix of the differences in fluorescence intensity before and after the addition of animal sera (0.10 vol%) to the AIE-polymers in two independent experimental batches (Ex1 and Ex2). The rightmost column indicates whether the data in the corresponding row was used as training data (denoted by "–") or test data (results of the verification are shown) in the holdout validation.

Dataset S4 (separate file). Data set matrix of the differences in fluorescence intensity before and after the addition of different quantities of FBS (0.10 vol%) to the AIE-polymer. The rightmost column indicates whether the data in the corresponding row was used as training data (denoted by "–") or test data (results of the verification are shown) in the holdout test.

Dataset S5 (separate file). Data set matrix of the differences in fluorescence intensity before and after the addition of FBS (0.10 vol%) stored under eight different conditions to the AIE-polymers. The rightmost column indicates whether the data in the corresponding row was used as training data (denoted by "–") or test data (results of the verification are shown) in the holdout test.

Dataset S6 (separate file). Data set matrix of the differences in fluorescence intensity before and after the addition of cardiomyocyte differentiation media (0.50 vol%) containing N2/B27 supplements treated under five different conditions to the AIE-polymers. The rightmost column indicates whether the data in the corresponding row was used as training data (denoted by "–") or test data (results of the verification are shown) in the holdout test.

Dataset S7 (separate file). Data set matrix of the differences in fluorescence intensity before and after the addition of supplements (0.02 vol%) stored for different periods to the AIE-polymers.

Dataset S8 (separate file). Data set matrix of the differences in fluorescence intensity before and after the addition of microbial culture media (1.0 vol%) to the AIE-polymers.

Dataset S9 (separate file). Data set matrix of the differences in fluorescence intensity before and after the addition of the AIE-polymers to yeast extracts (1.0 mg/mL) from two different manufacturers subjected to autoclave treatment for four different durations.

References

1 S. Tomita, H. Kusada, N. Kojima, S. Ishihara, K. Miyazaki, H. Tamaki and R. Kurita, *Chem. Sci.*, 2022, **13**, 5830–5837.
2 T. Wakimizu, K. Morikawa, K. Fukumura, T. Yuki, T. Adachi, Y. Kurata, J. Miake, I. Hisatome, M. Tsuneto and Y. Shirayoshi, *Regen. Ther.*, 2022, **21**, 239–249.
